# Epidemiological characteristics and hospital outcomes of hospitalized Lassa fever cases during the 2022-2023 outbreak in Liberia

**DOI:** 10.12688/f1000research.150743.1

**Published:** 2024-06-19

**Authors:** Emmanuel Dwalu, Hannock Tweya, Mher Beglaryan, Chukwuma D. Umeokonkwo, Ralph W. Jetoh, Bode I. Shobayo, Fahn Tarweh, Philip Owiti, Pryanka Relan, Shermarke Hassan, George W. Goteh, Darius B. Lehyen, Louis Ako-Egbe, Ibrahim Franklyn Kamara, Godwin E. Akpan, Peter Adewuyi, Patrick N. Kpanyen, Benjamin T. Vonhm, Julius S. M. Gilayeneh

**Affiliations:** 1 National Public Health Institute of Liberia, Monrovia, Liberia; 2 International Training and Education Center for Health (I-TECH), Lilongwe, Malawi; 3 Tuberculosis Research and Prevention Centre, Yerevan 0014, Armenia; 4 African Field Epidemiology Network, Kampala, Central Region, Uganda; 5 Ministry of Health, Nairobi, Kenya; 6 Health Emergencies Programme, World Health Organization, Geneva, Switzerland; 7 Infectious Diseases Data Observatory, University of Oxford, Oxford, UK; 8 World Health Organization Country Office, Monrovia, Liberia; 9 Reproductive Maternal Newborn Child and Adolescent Health Unit, World Health Organization Country Office, Freetown, Sierra Leone; 10 African Field Epidemiology Network, Monrovia, Liberia

**Keywords:** Liberia, Lassa fever, positivity rate, case fatality rate, outbreak, Integrated Disease Surveillance and Response, SORT IT, operational research

## Abstract

**Background:**

Lassa fever is an endemic and immediately notifiable disease in Liberia, and one laboratory confirmed case constitutes an outbreak. We described the epidemiological characteristics and hospital outcome of Lassa fever cases hospitalized during the 2022-2023 outbreak in Liberia.

**Methods:**

A cohort study was conducted using routine Lassa fever surveillance data from the 2022-2023 outbreak in Liberia. Descriptive statistics were used to summarize the data and log binomial regression to assess the association between epidemiological characteristics and mortality.

**Results:**

A total of 439 suspected Lassa fever cases were reported. The median age was 22 (interquartile range: 10-33) years and 233 (53%) were women. The median number of days between symptom onset and admission was 4 (IQR 2-7). Of the 439 cases, 416 (95%) were tested for Lassa fever and 138 were confirmed with 33% positivity rate. The majority, 290 (69%), of confirmed cases were <30 years, 78 (57%) were females, and 81 (59%) were reported during the dry season (October – March). Contact with rodents, 94 (68%), was the commonest mode of exposure. Fever, 128 (93%), malaise, 121 (88%), headache, 114 (83%) and myalgia, 114 (83%) were the most common clinical characteristics. There were 83 (19%) deaths among hospitalized suspected Lassa fever cases - 42 deaths (15%) among 278 individuals who tested negative and 41 among confirmed cases with 30% case fatality rate (CFR). The highest CFR was recorded among those aged 40-49 years, 8 (67%) and those aged≥50, 5 (63%). There was no significant association between epidemiological characteristics and Lassa fever mortality.

**Conclusions:**

The outbreak highlighted a high disease burden of Lassa fever with young adults disproportionately infected, and substantial mortality, even among those who tested negative for the virus. This underscores the urgent need for preventive measures like vaccines and health education campaigns.

## Introduction

Lassa fever (LF) is a viral haemorrhagic fever caused by the Lassa virus, a member of arenavirus group. ^1^^–^^3^ This zoonotic disease is primarily transmitted to humans through direct or indirect contact with the urine or feces of the infected Mastomys rats (Mastomys natalensis) during hunting or processing for consumption. ^4^^–^^6^ The rats are the natural reservoir for the Lassa fever virus. Additionally, the virus may be transmitted from human to human through contact with an infected person's blood, faeces, or other bodily secretions. ^7^^,^^8^ Pregnant women and children have the highest risk of contracting the disease. ^3^^,^^9^ The incubation period for Lassa fever ranges from 2-21 days. A systematic review conducted in 2021 reported on clinical data, identifying fever (88%), headache (50%), vomiting (49%), abdominal pain (42%), and cough (35%) as the most frequently presenting symptoms in individuals suspected of having Lassa fever. The disease is diagnosed through a real-time polymerase chain reaction (RT-PCR) test and intravenous (IV) ribavirin is a recommended treatment. ^10^^,^^11^ Additionally, oral ribavirin can be used for post-exposure prophylaxis. However, recent studies have reported contradictory results regarding the effectiveness of ribavirin, prompting need for further investigation. ^12^

Lassa fever is a significant global public health challenge, recognized by the World Health Organisation (WHO) as a priority disease for surveillance, research and vaccine development efforts. ^1^^,^^11^ Endemic to various West African countries, including Guinea, Liberia, Nigeria, and Sierra Leone, ^13^ the disease is estimated to cause 100,000 to 300,000 new cases and 5,000-10,000 deaths annually. ^6^^,^^14^ Lassa fever exhibits a seasonal pattern, with most cases reported during the dry season each year. ^3^^,^^15^ The case fatality rate (CFR) among hospitalized patients is estimated to be between 15% and 20% but can reach 50% during epidemics. ^7^

In Liberia, Lassa fever is a notifiable disease and one laboratory confirmed case constitutes an outbreak. Liberia is experiencing its most protracted Lassa fever outbreak, which started in January 2022. ^16^ In June 2021, the National Integrated Disease Surveillance and Response (IDSR) Technical Guidelines were revised, incorporating an updated Lassa fever case definition. This coincided with efforts to improve case detection through healthcare worker training. ^17^ Despite these measures, crucial information such as the positivity rate, socio-demographic and clinical characteristics, mode of exposure to rodents, and CFR regarding the current outbreak remains largely unknown. Therefore, this study aimed to understand the 2022-2023 Lassa fever outbreak in Liberia by investigating characteristics of suspected and confirmed Lassa fever cases, mode of exposure, healthcare access (i.e. timelines of admission and treatment) and hospital outcomes.

## Methods

### Study design

We conducted a cohort study in Liberia using routine national outbreak surveillance data of all suspected Lassa fever cases reported between January 2022 and December 2023.

### Study setting

***General setting***

Liberia has a population of over five million people. The country has a dry season (late October to March) and a rainy season (April to early October). Its capital city is Monrovia and the country is divided into 15 administrative units called counties, five health regions each comprising 3 counties, and 98 health districts. ^18^ The Liberian healthcare system operates at five levels: national, county, district, health facility, and community. There is a 3-tier system for service delivery: a primary level (primary health clinics), a secondary level (health center and county/regional hospitals) and a tertiary level (national specialized hospitals). Each level is staffed with designated healthcare workers including surveillance officers who coordinate disease routine surveillance activities. Liberia has 962 functional health facilities categorized as primary-level clinics, secondary-level health centers, and tertiary-level hospitals. The county hospitals have dedicated Lassa fever treatment centres. Health care services in public health facilities in Liberia are provided free of charge.

***Specific setting***

Case detection for Lassa fever began at the community or health facility level. At the community level, community health workers identified potential Lassa fever cases. Potential cases were referred to the nearest health facility for verification. At the health facility level, clinicians used the case definition for Lassa fever to suspect cases based on symptoms. A suspected case was defined as a patient experiencing a fever lasting for 2-21 days (above 38°C) with one or more additional symptoms such as malaise, headache, or muscle pain. Alternatively, patients who had not responded to anti-malarial treatment within 48-72 hours or had a history of rodent contact were also considered suspected cases. ^17^ Exposure to Lassa virus was defined to encompass any contact with rodents, either directly (handling rodents or their food sources) or indirectly (through contaminated utensils, feces, urine, or consumption of rodent-borne food). Additionally, exposure included contact with bodily fluids from a person infected with the Lassa fever virus. A probable case was defined as a suspected case who has one or more of the following complications: hearing loss, facial or neck swelling, seizures/convulsions, restlessness, confusion, hypotension, abdominal bleeding. Once verified, demographic and clinical information of the suspected cases was recorded in the IDSR ledger. The IDSR case alert and laboratory submission forms were then completed and finally samples were collected.

Samples were sent to the National Public Health Reference Laboratory (NPHRL) to confirm Lassa fever. Laboratory confirmation was performed by RT-PCR using a Real Star Lassa Virus RT-PCR kit. A confirmed case was defined as a suspected or probable case with a confirmed/positive laboratory test (positive IgM antibody, positive PCR or virus isolation) or epidemiologically linked to a laboratory confirmed case. ^19^ Once results were available, the NPHRL notified the National Public Health Institute of Liberia (NPHIL) and county teams. The county health teams, facilitated by the county surveillance officers (CSO), then sent the results to the district level through the district surveillance officers (DSO) who notified the health facilities.

While awaiting the laboratory test results, the suspected cases were isolated and admitted to a treatment centre where IV Ribavirin treatment was initiated. Daily assessments were performed using charts to monitor the patients’ progress. On average, confirmed Lassa fever cases were treated with IV Ribavirin for 10 days depending on the patient's clinical evolution and recovery. Individuals with negative results were discharged immediately after the Ribavirin treatment was discontinued.

The data from the IDSR ledger and case alert and laboratory paper-based forms were regularly entered into an MS Excel dataset containing the line-list of Lassa fever surveillance data in each district, then submitted to the county level and onward to the National Public Health Institute of Liberia.

### Study population

All suspected cases reported during the outbreaks in Liberia between January 2022 and December 2023 were included in the analysis.

### Study data sources, variables, and validation

We obtained data on Lassa fever cases from the Microsoft Excel national surveillance database maintained by the Division of Infectious Disease and Epidemiology, National Public Health Institute of Liberia, for the period 2022-2023. Due to incomplete information in some records we reconciled the data using the county surveillance database and selected treatment centers.

### Statistical analysis

We performed all statistical analyses using Epi Info analysis software (version 7.2.5.0.) and Stata v18.0 (open source alternative is R: The R Project for Statistical Computing), version 4.3.2. Descriptive statistics were used to summarize the data. Categorical variables, such as sex, county, and symptoms, were presented as frequencies and proportions. Continuous variables including age, time from symptom onset to admission and to ribavirin treatment were presented as medians with interquartile range (IQR). We estimated positivity rate and case fatality of Lassa fever: positivity rates were calculated by dividing the number of confirmed cases by the total number of individuals tested while the case fatality rate (CFR) was calculated by dividing the number of deaths by the total number of confirmed Lassa fever cases. The confirmed Lassa fever cases were plotted by county using Arc Geographic Information System Pro ( ArcGIS Pro 3.2.2) (open source alternative: QGIS, version 3.36.3) to visualize the geospatial distribution of the disease. An epidemic curve was constructed using the date of symptom onset and mortality for all confirmed cases. We used log binomial regression to assess the association between socio-demographics, clinical characteristics, and case fatality rates. Models’ results were presented as Risk Ratios (RR) with 95% confidence intervals (CI) and p-values <0.05 were considered statistically significant.

## Results

### Characteristics of the suspected lassa fever cases

**Figure 1.**
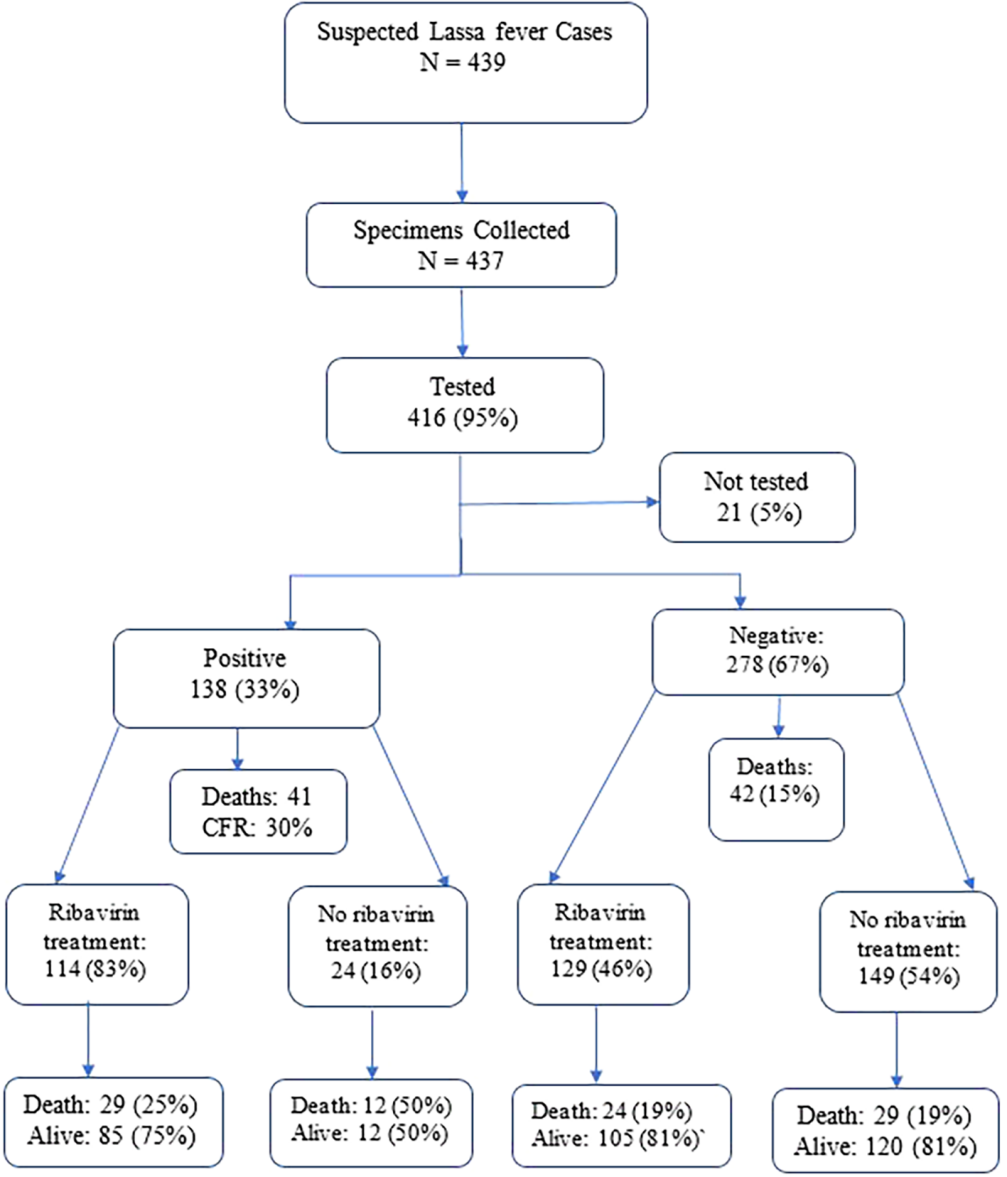
Lassa fever diagnosis, treatment and hospital outcome, Liberia 2022-2023.

A total of 439 suspected Lassa fever cases were reported between January 2022 and December 2023 ( Figure 1). The median age was 22 (IQR 10-33) years. The majority (66%, 290/439) of the suspected cases were below 30 years There were more women (53%, 233/439) than men. Occupation data was missing for 174 participants. Among the 265 with recorded occupations, students constituted the largest group (32%, 141/439) followed by business persons (12%, 51/439). Geographically, Bong County reported the highest number of suspected cases (44%, 192/439), followed by Grand Bassa County (20%, 89/439) and Nimba County (18%, 80/439) ( Table 1).

**Table 1.** Characteristics and positivity rate for Lassa fever diagnosis using reverse transcription-polymerase chain reaction (RT-PCR), Liberia, 2022-2023.

Characteristics	Total suspected *		PT-PCR tested *		Positivity rate ^¥^	
	n	%	n	%	n	%
**Total**	439		416	95	138	33
Age (years)						
≤14	143	33	133	32	45	33
15-29	147	33	140	34	50	36
30-39	78	18	73	18	23	31
40-49	46	10	45	11	12	27
≥50	25	6	25	6	8	32
Median age	22 (IQR:10-33) Years					
**Gender**						
Male	206	47	206	50	60	29
Female	233	53	233	56	78	33
**Occupation**						
Business	51	11	51	12	15	29
Farmer	33	8	31	7	8	26
Housewife	16	4	16	4	8	50
Student	141	32	137	33	50	36
Tapper	19	4	19	5	11	58
Others ^β^	8	2	8	2	3	38
Unknown	171	39	154	37	13	8
**Reporting county**						
Bong	192	44	171	41	60	35
Grand Bassa	89	20	88	21	45	51
Nimba	80	18	80	19	27	34
Montserrado	56	13	56	13	5	9
Others ^β^	22	5	21	5	1	5

* Number (and column-proportion) for characteristics and RT-PCR tested.

^¥^ Number (and row-proportion) positivity rate).

^β^ Others (number of suspected cases): River Gee (n=5), Grand Kru (n=7), Maryland (n=3), Bomi (n=2), Lofa (n=2), Margibi (n=2), Sinoe (n=1).

### Time from symptom onset to hospital admission

Among 439 individuals, about one quarter (26%, 116/439) were excluded due to missing data on date of symptom onset or admission ( Table 2). Among the 323 participants with recorded dates, the overall median number of days between symptom onset and admission was 4 (IQR 2-7). Median times by gender were similar (4.5 (IQR: 2-7) vs 4.0 (IQR 1-7) days). When stratified by county, Nimba had the shortest median time, with a median of <1 day (IQR < 1-1.5).

**Table 2.** Time from Symptom Onset to Hospital Admission among suspected Lassa fever cases, Liberia, 2022-2023.

	Total	Symptom onset to admission		
		n (%)	Median (IQR) *	
**Overall**	**439**	**323 (74)**	**4**	**(2-7)**
Age (years)				
<=14	143	103 (72)	3	(2-6)
15-29	147	121 (82)	5	(1-7)
30-39	78	52 (67)	4.5	(2-6.5)
40-49	46	32 (70)	4	(1.5-7)
50+	25	15 (60)	2	(0-8)
Gender				
Female	233	139 (60)	4.5	(2-7)
Male	206	174 (85)	4	(1-7)
County				
Bong	192	108 (56)	5	(3-7)
Grand Bass	89	87 (98)	3	(2-7)
Nimba	80	76 (95)	0	(0-1.5)
Montserrado	56	52 (93)	5	(2-7)
Other ^β^	0	-	-	-

* IQR=Interquartile range.

^β^ Others (number of suspected cases): River Gee, Grand Kru, Maryland, Bomi, Lofa, Margibi, Sinoe.

### Positivity rate of lassa fever cases

**Figure 2.**
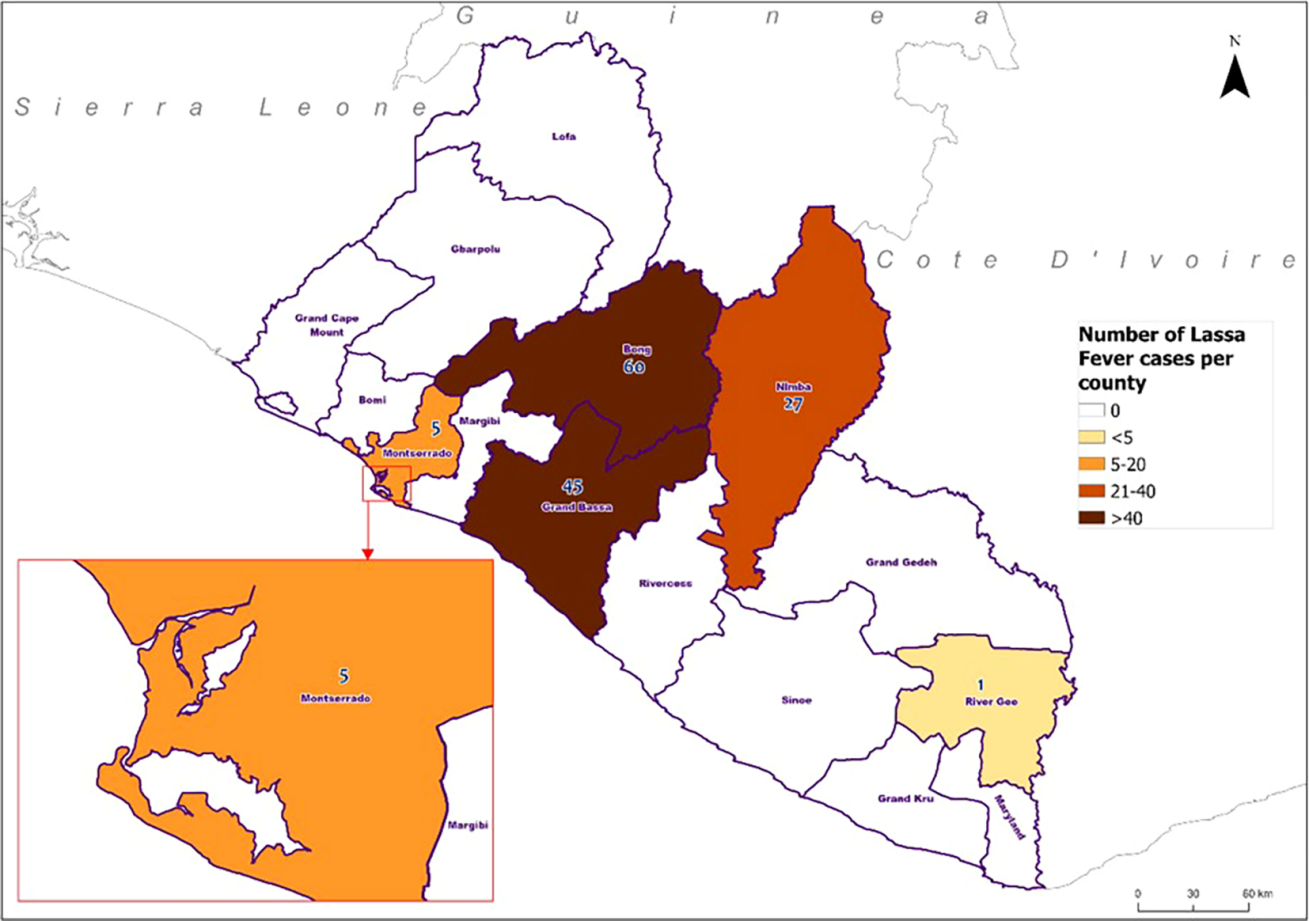
Geographical distribution of confirmed Lassa fever cases by reporting county, Liberia, 2022-2023.

Of the 439 individuals suspected of Lassa fever, 437 specimens were collected and majority (95%, 416/439) were tested using RT-PCR test and 138 were Lassa fever positive. The overall positivity rate was 33% (138/416) ( Figures 1 & 2; Table 1). Among suspected cases with recorded occupations, tappers had the highest positivity rate (58%, 11/19), followed by housewives (50%, 8/16). Positivity rates were highest in Grand Bassa County (51%, 45/88), followed by Bong (35%, 60/171) and Nimba Counties (34%, 27/80).

### Ribavirin treatment among lassa fever cases

Ribavirin treatment was administered to 57% (249/439) of hospitalized suspected Lassa fever cases ( Figure 1).

Ribavirin treatment was administered to 83% (114/138) of the confirmed Lassa fever cases and 46% (129/278) of those with negative PCR results ( Figure 2).

### Epi-curve of confirmed Lassa fever cases by reporting epi week and year

**Figure 3.**
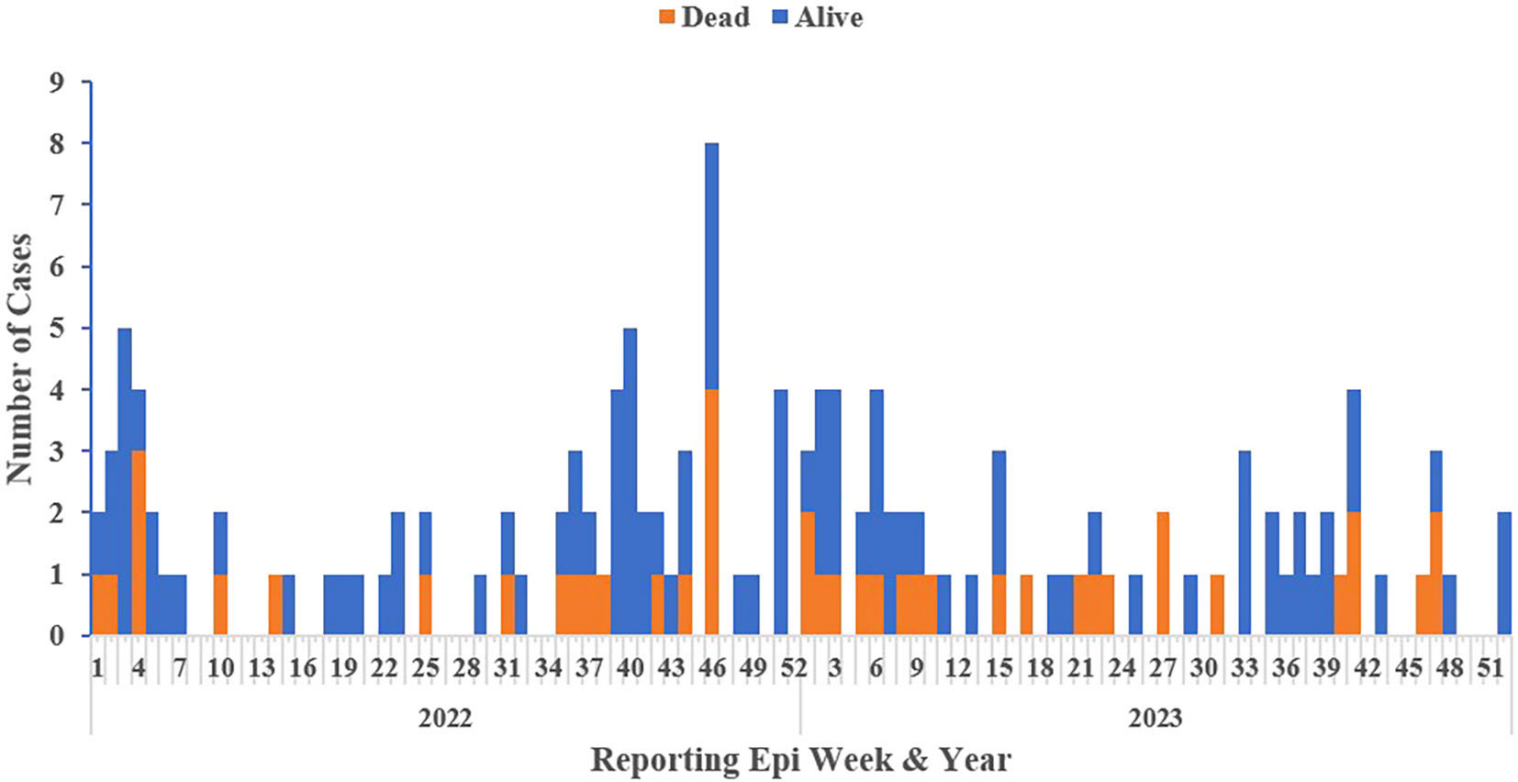
Epidemic curve of confirmed Lassa fever cases and deaths by reporting week and year, Liberia, 2022-2023.

The 2022 calendar year had more confirmed cases, accounting for over half (52%, 72/138) of the reported cases. More cases (59%, 81/138) clustered between epi-weeks 1-12 and 40-52, aligning with the dry season (October – March) ( Figure 3).

### Type of exposure and clinical characteristics among confirmed Lassa fever cases

Rodent exposure was the most common type of contact (68%, 94/138) among confirmed Lassa fever cases. The most common clinical characteristic was fever (93%, 128/138), followed by malaise (88%, 121/138), headache (83%, 114/138), and myalgia (83%, 114/138). Less than 1% of patients reported hearing loss ( Table 3).

**Table 3.** Exposure and clinical characteristics in confirmed Lassa fever cases, Liberia, 2022-2023 (N=138).

Characteristics	Confirmed N=138	
Type of exposure	n	%
Exposure to rodent	95	68
Contact with confirmed Lf case	11	8
Unknown	32	24
Signs and symptoms (n=138)		
Fever >38 °C	128	93
Malaise	121	88
Myalgia	114	83
Headache	114	83
Sore throat	86	62
Diarrhea	85	62
Red eyes	81	59
Vomiting	77	56
Chest pain	76	55
Nausea	70	51
Cough	55	40
Bleeding	46	33
Swollen face	43	31
Swollen neck	26	19
Hearing loss	1	0.7

### Mortality and case fatality rates

There were 83 (19%) deaths among hospitalized suspected Lassa fever cases; 41 were among confirmed cases, with a 30% CFR. The remaining 42 deaths (15%) occurred among 278 individuals who tested negative ( Figure 1, Tables 1 & 4). The CFR varied significantly by age group (p-value=0.006). The highest CFR was observed in individuals aged 40-49 years (67%, 8/12), followed by those aged ≥50 years (63%, 5/8). Lower CFRs were seen in the ≤14-year-old group (27%, 12/45), the 30-39year old group (26%, 6/23), and the 15-29year old group (20%, 10/50).

**Table 4.** Hospital outcome and factors associated with mortality among confirmed Lassa fever cases, Liberia, 2022-2023.

Variable	Confirmed (N=138)	Deaths (CFR (%) (N=41)	cRR (95% CI)	P value	aRR (95% CI)	P value
**Age (Yrs)**	N	N (%)				
<30	95	22 (23)	Ref			
>=30	43	19 (44)	1.9 (1.160- -3.136	0.011	1.2 (0.554-2.656)	0.628
**Sex**						
Male	60	20 (33)	1.2 (0.742--2.065)	0.413		
Female	78	21 (27)	Ref			
**Reporting County**						
Bong	60	14 (23)	Ref			
Grand Bassa	45	11 (24)	1.0 (0.526-2.085)	0.895		
Nimba	27	13 (48)	2.0 (1.129- -3.771)	0.019	1.1 (0.357-3.382)	0.868
**Occupation (n=95)**						
Business	15	6 (40)	Ref			
Farmer	8	5 (63)	1.5 (0.688-3.547)	0.286		
Healthcare workers	3	0				
House wife	8	2 (25)	0.6 (0.161-2.412)	0.495		
Student	50	10 (20)	0.5 (0.217-1.148)	0.102	0.6 (0.240-2.020)	0.506
Tapper	11	4 (36)	0.9 (0.335-2.465)	0.851		
**Duration_ onset to admission (n=103)**						
<7 days	59	19 (32)	Ref			
>=7 days	44	33 (75)	0.7 (0.412-1.460)	0.432		

*CFR=case fatality rate;

cRR=crude risk ratio;

aRR=adjusted risk ratio;

CI=confidence interval;

Ref=reference point.

### Factors associated with mortality among confirmed lassa fever cases

At bivariate level, we found significant associations between age ≥30 (cRR=1.9, 95% CI=1.160-3.136, p=0.011), reporting county (Nimba (cRR=2.0, 95% CI=1.129-3.771, p=0.019) and Lassa fever mortality. When modelled using multiple logistic regression, none of these associations were significantly associated with Lassa fever mortality among confirmed cases ( Table 4).

## Discussion

The study described the epidemiological characteristics and case fatality rates among Lassa fever cases hospitalized during the 2022-2023 outbreak in Liberia. Findings showed a substantial burden of Lassa fever in Liberia during the outbreak with high positivity rates (33%) and case fatality rates (30%), with an even higher CFR among the elderly population. Younger age groups were disproportionately affected. Furthermore, the dry season coincided with a surge in confirmed cases. Fever, headache and malaise were the most frequently reported symptoms, and rodent exposure was highest among the confirmed cases. Surprisingly, a high mortality rate (13%) was observed among hospitalized individuals without Lassa fever, highlighting challenges in diagnosis and in Lassa fever management.

Similar to other studies, ^6^^,^^19^ the study found high testing and positivity rates. The high testing and positivity could be because Lassa fever is one of the immediately reportable priority diseases under surveillance in Liberia and an endemic disease. Additionally, strengthened surveillance systems at all healthcare delivery levels with assigned surveillance officers for the detection and reporting of suspected Lassa fever, a functional testing national public health reference laboratory and, most importantly, the revised 2021 case definition of Lassa fever all contributed to improved detection of Lassa fever. ^17^

The study highlights that younger age groups are disproportionately affected by Lassa fever. This vulnerability is likely to be due to increased exposure to rodent reservoirs. Most confirmed cases reported direct or indirect contact with rodents suggesting exposure to rodent droppings or urine during outdoor activities and food handling. Similar to other studies in Liberia ^6^^,^^19^ and Nigeria, ^20^ most of the cases were recorded in the dry season (the latter and earlier parts of the two years in the study period: October to March) with 2023 accounting for the highest burden. The seasonality of Lassa fever cases in West Africa's dry season is likely due to a combination of factors related to the behavior of the main reservoir, the Mastomys natalensis rodent. During the dry season, with less vegetation and food sources outside, these rodents are more likely to seek shelter and food inside homes, increasing their contact with humans. This could lead to more frequent interactions between rodents, potentially increasing the spread of the Lassa fever virus within the rodent population. Less plant cover during the dry season could make rodent burrows and movements more noticeable for hunting. A better cleaning environment both in the house and the outside environment might help to reduce the contact between rats and humans. Additionally, effective community infection prevention control programmes in endemic areas might be of help.

Our study identified a high CFR of 33%, more than double the 15% reported by the WHO for the region. While this CFR is lower than those reported in Nigeria (60%) ^21^ and Sierra Leone (69%) ^22^ and slightly lower than a previous study in Liberia (40%), it remains a significant cause for concern. Furthermore, high CFRs were observed in both treated and untreated patients. The short time between symptom onset and hospital admission suggests that healthcare-seeking behaviors alone cannot fully explain the high CFR. Previous studies have raised concerns about the effectiveness of ribavirin treatment and its potential harm. ^12^ The reliability of the human clinical trial data supporting ribavirin's use has also been questioned, with pre-clinical studies suggesting that current dosing regimens may not reliably inhibit Lassa fever virus replication. ^12^ Similar to other studies, the CFR was higher among elderly people. However, the reasons for this are unclear, warranting further research in understanding the occurrence. Surprisingly, CFR was higher in Nimba, an endemic Lassa fever county, a finding similar to what was reported in a previous study. ^6^ The high CFR in Nimba could be due to the fact that the county is endemic for Lassa fever, with the majority of cases detected at the health facility rather than through early community detection, which impacts on early treatment. The study highlights challenges in Lassa fever management. Suspected Lassa fever cases are admitted and begin ribavirin while waiting for test results. Some of the Lassa fever symptoms (fever and headache) overlap with common diseases like malaria, leading to unnecessary admissions and ribavirin use as well as delayed appropriate treatment. This is concerning, as a 13% mortality rate was observed among those who did not have Lassa fever which may be due to other undiagnosed infections. While the strategy aims to combat Lassa fever’s high CFR, improvements in diagnosis and management are crucial.

These findings have an important implication for the programme’s practice and policy. Addressing the screening and management challenges surrounding Lassa fever requires a proactive shift towards preventive measures. Mass vaccination campaigns, although in their developmental stages, offer a promising avenue for mitigating the burden of Lassa fever. Initiatives by the WHO and Coalition for Epidemic Preparedness Innovations (CEPI) prioritize Lassa fever for vaccine development and enhanced surveillance efforts, ^23^ signaling a step in the right direction. However, given the time frame for vaccine development and implementation, immediate action is warranted. Based on a clearer understanding of seasonal variations within Liberia and across West Africa, public health interventions like vector control programmes and year-round community education campaigns, intensified during the dry season when Lassa fever transmission peaks, can significantly enhance community awareness and prevention, thereby reducing mortality rates and easing the burden on healthcare systems. Implementing rodent-proofing measures can further reduce exposure risks.

Our findings should be viewed with the following limitations: Firstly, there were incomplete data on duration from onset of symptoms to hospital admission. Exclusion of these records might have affected the estimation of the time from onset of symptoms to hospital admission with respect to socio-demographic characteristics. Secondly, our data was focused on facility-based rather than community-based surveillance, meaning that those who did not seek healthcare were not captured, and we might therefore have underestimated the number of Lassa fever cases. Despite these limitations, the study used surveillance data which reflect the program setting, making the findings useful to inform policy and programmes in Liberia and other comparable settings to prevent outbreaks of Lassa fever.

## Conclusions

This study showed a significant burden of Lassa fever in Liberia during the 2022-2023 outbreak, characterized by high positivity, high CFR, and a substantial mortality among those without the disease, highlighting the urgent need for proactive prevention measures such as vaccination campaigns and intensive public education. Furthermore, the CFR was high among the elderly population, warranting further investigation. Additionally, the incompleteness of some records highlights the need to strengthen data collection practices within healthcare facilities to ensure complete and accurate data for informed outbreak response efforts.

### Ethics consideration

Access to data for this study was granted by the National Public Health Institute of Liberia. Ethics approvals were obtained from University of Liberia Ethics Review Board on September 27, 2023 (protocol# 23-09-390) and the Ethics Advisory Group for the International Union Against Tuberculosis and Lung Disease, Paris, France, on August 9, 2024; (EAG# 24/23). Informed consent was not obtained as we used routine programme data, which was anonymized by delinking patient identifiers from the dataset.

## Author contributions

“Conceptualization, E. D, R.W.J, B.I.S, H. T, F.T, P. O, M.B, I.F.K, G.W.G, P. K, D. B. L, L.A.E, P. R, S.H; B.T.V., J.S.M.G; methodology, E. D, H.T, P. O, M.B, C.D.U, R.W.J, B.I.S, F. T, P. A, G.W.G,D.B.L, L.A.E, P. R, S. H, B.T.V, P. K, P.A., I.F.K, J.S.M.G; software; validation, E. D, G.W.G; formal analysis, E. D, H.T, M. B, C.D.U, P.A., GEA; investigation; resources; data curation; writing—original draft preparation, E. D, H.T, M. B, writing—review and editing, E. D, H.T, F. T, P.O, C.D.U, B.T.V, M. B, B.I.S, P. K, P. A, L.A.E, P. R, S. H, I.F.K, GEA; visualization, G.E.A, E.D; supervision; project administration; funding acquisition. All authors have read and agreed to the last version of the manuscript.”

## Open access statement

In accordance with WHO’s open-access publication policy for all work funded by WHO or authored/co-authored by WHO staff members, WHO retains the copyright of this publication through a Creative Commons Attribution IGO license ( http://creativecommons.org/licenses/by/3.0/igo/legalcode) which permits unrestricted use, distribution and reproduction in any medium provided the original work is properly cited.

## Data Availability

The dataset used for this study is available at the Division of Infectious Disease and Epidemiology, National Public Health Institute of Liberia and can be accessed upon request in line with the existing data request guide, which provides opportunity for both internal and external data requests ( https://www.nphil.gov.lr/wp-content/uploads/2024/03/nphil-data-request-guide.pdf).
