# Epidemiological characteristics and hospital outcomes of hospitalized Lassa fever cases during the 2022-2023 outbreak in Liberia

**DOI:** 10.12688/f1000research.150743.3

**Published:** 2025-06-10

**Authors:** Emmanuel Dwalu, Hannock Tweya, Mher Beglaryan, Chukwuma D. Umeokonkwo, Ralph W. Jetoh, Bode I. Shobayo, Fahn Tarweh, Philip Owiti, Pryanka Relan, Shermarke Hassan, George W. Goteh, Darius B. Lehyen, Louis Ako-Egbe, Ibrahim Franklyn Kamara, Godwin E. Akpan, Peter Adewuyi, Patrick N. Kpanyen, Benjamin T. Vonhm, Julius S. M. Gilayeneh

**Affiliations:** 1 National Public Health Institute of Liberia, Monrovia, Liberia; 2 International Training and Education Center for Health (I-TECH), Lilongwe, Malawi; 3 Tuberculosis Research and Prevention Centre, Yerevan 0014, Armenia; 4 African Field Epidemiology Network, Kampala, Central Region, Uganda; 5 Ministry of Health, Nairobi, Kenya; 6 Health Emergencies Programme, World Health Organization, Geneva, Switzerland; 7 Infectious Diseases Data Observatory, University of Oxford, Oxford, UK; 8 World Health Organization Country Office, Monrovia, Liberia; 9 Reproductive Maternal Newborn Child and Adolescent Health Unit, World Health Organization Country Office, Freetown, Sierra Leone; 10 African Field Epidemiology Network, Monrovia, Liberia

**Keywords:** Liberia, Lassa fever, positivity rate, case fatality rate, outbreak, Integrated Disease Surveillance and Response, SORT IT, operational research

## Abstract

**Background:**

Lassa fever (LF) is an endemic and immediately notifiable disease in Liberia, and one laboratory confirmed case constitutes an outbreak. We described the epidemiological characteristics and hospital outcomes of LF cases hospitalized during the 2022-2023 outbreak in Liberia.

**Methods:**

A retrospective cohort study was conducted using routine LF surveillance data from the 2022-2023 outbreak in Liberia. Descriptive statistics were used to summarize the data and log binomial regression to assess the association between epidemiological characteristics and mortality.

**Results:**

A total of 439 suspected LF cases were reported. The median age was 22 (interquartile range (IQR): 10-33) years and 233 (53%) were females. The median number of days between symptom onset and admission was 4 (IQR 2-7). Of the 439 cases, 416 (95%) were tested for LF and 138 were confirmed with 33% positivity rate. The majority, 95 (69%), of confirmed cases were <30 years, 78 (57%) were females, and 81 (59%) were reported during the dry season (October – March). Contact with rodents, 95 (69%), was the commonest mode of exposure. Fever, 128 (93%), malaise, 121 (88%), headache, 114 (83%) and myalgia, 114 (83%) were the most common clinical presentations. There were 83 (19%) deaths among hospitalized suspected LF cases - 42 deaths (15%) among 278 individuals who tested negative and 41 among confirmed cases with 30% case fatality rate (CFR). Presenting CFR per age group, age 40-49 years accounted for 8/12 (67%) and those aged≥50 reported 5/8 (63%) of the deaths among the confirmed cases. There was no significant association between epidemiological characteristics and LF mortality.

**Conclusions:**

The outbreak highlighted a high disease burden of LF with young adults disproportionately infected, and mortality, even among those who tested negative for the virus. This underscores the urgent need for preventive measures like vaccines and health education campaigns.

## Introduction

Lassa fever (LF) is a viral haemorrhagic fever caused by the Lassa virus (LASV), a member of arenavirus group. ^1^^–^^3^ This LASV would be primarily transmitted to humans through direct or indirect contact with the urine or feces of the infected natal mastomys mice (Mastomys natalensis) during hunting or processing for consumption. ^4^^–^^6^ The mice are the natural reservoir for the LASV. Additionally, the virus may be transmitted from human to human through contact with an infected person's blood, faeces, or other bodily secretions. ^7^^,^^8^ Pregnant women and children have the highest risk of contracting the disease. ^3^^,^^9^ The incubation period for LF ranges from 2-21 days. A systematic review conducted in 2021 reported on clinical data, identified fever, headache, vomiting, abdominal pain, and cough as the most frequently presenting symptoms in individuals suspected of having LF and a retrospective study in 2022 identified fever, fatigue, sore throat, loss of appetite, headache, vomiting, and myalgia as presenting symptoms among confirmed cases. The disease is diagnosed through a real-time polymerase chain reaction (RT-PCR) test and intravenous (IV) ribavirin is a recommended treatment. ^10^^,^^11^ Additionally, oral ribavirin can be used for post-exposure prophylaxis. However, recent studies have reported contradictory results regarding the effectiveness of ribavirin, prompting need for further investigation. ^12^

LF is a significant global public health challenge, recognized by the World Health Organization (WHO) as a priority disease for surveillance, research and vaccine development efforts. ^1^^,^^11^ Endemic to various West African countries, including Guinea, Liberia, Nigeria, and Sierra Leone, ^13^ the disease is estimated to cause 100,000 to 300,000 new cases and 5,000-10,000 deaths annually. ^6^ LF exhibits a seasonal pattern, with most cases reported during the dry season each year. ^3^^,^^14^ The case fatality rate (CFR) among hospitalized patients is estimated to be between 15% and 20% but could be more during epidemics. ^7^

In Liberia, LF is a notifiable disease and one laboratory confirmed case constitutes an outbreak. Liberia is experiencing its most protracted LF outbreak, which started in January 2022. ^15^ In June 2021, the National Integrated Disease Surveillance and Response (IDSR) Technical Guidelines were revised, incorporating an updated Lassa fever case definition. This coincided with efforts to improve case detection through healthcare worker training. ^16^ Despite these measures, crucial information such as the positivity rate, socio-demographic and clinical characteristics, mode of exposure, CFR, and factors associated with mortality in this outbreak remain largely unknown. Therefore, this study aimed to understand the 2022-2023 LF outbreak in Liberia by investigating characteristics of suspected and confirmed LF cases, mode of exposure, healthcare access (i.e. timelines of admission and treatment), hospital outcomes, and factors associated with mortality among confirmed LF patients.

## Methods

### Study design

We conducted a retrospective cohort study in Liberia using routine national LF outbreak surveillance data of all suspected LF cases reported between January 2022 and December 2023.

### Study setting

***General setting***

Liberia has a population of over five million people. The country has a dry season (late October to March) and a rainy season (April to early October). Its capital city is Monrovia and the country is divided into 15 administrative units called counties, five health regions each comprising 3 counties, and 98 health districts. ^17^ The Liberian healthcare system operates at five levels: national, county, district, health facility, and community. There is a 3-tier system for service delivery: a primary level (primary health clinics), a secondary level (health center and county/regional hospitals) and a tertiary level (national specialized hospitals). Each level is staffed with designated healthcare workers including surveillance officers who coordinate disease routine surveillance activities. Liberia has 962 functional health facilities categorized as primary-level clinics, secondary-level health centers, and tertiary-level hospitals. The county hospitals have dedicated LF treatment centres. Health care services in public health facilities in Liberia are provided free of charge.

***Specific setting***

Case detection for LF began at the community or health facility level. At the community level, community health workers identified potential LF cases using the community case definition. Potential cases were referred to the nearest health facility for verification using the community trigger and referral form. Referrals were made immediately or within 24 hours of identification, with a phone call or written message delivered to the nearby health facility. At the health facility level, clinicians/health facility surveillance focal persons used the standard case definition for LF to identify suspected cases based on symptoms. A suspected case was defined as a patient experiencing a fever lasting for 2-21 days (above 38°C) with one or more additional symptoms such as malaise, headache, or muscle pain. Alternatively, patients who had not responded to anti-malarial treatment within 48-72 hours or had a history of rodent contact were also considered suspected cases. ^16^ Exposure to LASV was defined to encompass any contact with rodents, either directly (handling rodents or their food sources) or indirectly (through contaminated utensils, feces, urine, or consumption of food contaminated with rodent feces or urine). Additionally, exposure included contact with bodily fluids from a person infected with the Lassa fever virus. A probable case was defined as a suspected case who has one or more of the following complications: hearing loss, facial or neck swelling, seizures/convulsions, restlessness, confusion, hypotension, abdominal bleeding. Once verified, demographic and clinical information of the suspected cases was recorded in the IDSR ledger. The IDSR case alert and laboratory submission forms were then completed and finally whole blood samples were collected.

Samples were sent to the National Public Health Reference Laboratory (NPHRL) to confirm LF. Laboratory confirmation was performed by RT-PCR using a Real Star LASV RT-PCR kit. A confirmed case was defined as a suspected or probable case with a confirmed/positive laboratory test (positive IgM antibody, positive RT-PCR or virus isolation) or epidemiologically linked to a laboratory confirmed case. ^18^ Once results were available, the NPHRL notified the National Public Health Institute of Liberia (NPHIL) and county teams. The county health teams, facilitated by the county surveillance officers, then sent the results to the district level through the district surveillance officers who notified the health facilities.

While awaiting the laboratory test results, individuals were isolated and admitted to a treatment centre where IV ribavirin treatment was initiated if they were classified as “suspected Lf patients”. Ribavirin was administered only to suspected LF cases while awaiting laboratory test results, and treatment was discontinued for individuals with negative results. Daily assessments were performed (i.e. routine examinations including physical exams, patients’ vitals, malaria test, and other necessary checks, etc.) using charts to monitor the patients’ progress. On average, confirmed LF cases were treated with IV ribavirin for 10 days depending on the patient's clinical evolution and recovery. Individuals with negative results after a RT-PCR testing results were discontinued from ribavirin treatment and were discharged home immediately.

The data from the IDSR ledger and case alert and laboratory submission paper-based forms were regularly entered into an MS Excel dataset containing the line-list of LF surveillance data in each district, then submitted to the county level and onward to the National Public Health Institute of Liberia.

### Study data sources, variables, and validation

We obtained data on LF cases from the Microsoft Excel national surveillance database maintained by the Division of Infectious Disease and Epidemiology, National Public Health Institute of Liberia, for the period 2022-2023. Due to incomplete information in some patient records submitted at the national level, we reconciled the data using the county surveillance database. We opted for four sites instead of all eight because these were the sites reporting majority of the cases and reviewing records from these sites would address the incomplete record issue or data quality issues in the existing data set used for the study.

### Statistical analysis

We performed all statistical analyses using Epi Info analysis software (version 7.2.5.0.) and Stata v18.0 (open source alternative is R: The R Project for Statistical Computing), version 4.3.2. The Epi Info analysis software was used for all descriptive statistics while the Stata v18.0 (open-source alternative is R: The R Project for Statistical Computing), was used to conduct the regression analysis. Descriptive statistics were used to summarize the data. Categorical variables, such as sex, county, and symptoms, were presented as frequencies and proportions. Continuous variables including age, time from symptom onset to admission and to ribavirin treatment were presented as medians with interquartile range (IQR). We estimated LASV positivity and case fatality of LF: LF positivity were calculated by dividing the number of confirmed cases by the total number of individuals tested while the CFR was calculated by dividing the number of deaths among confirmed LF cases by the total number of confirmed LF cases. The confirmed LF cases were plotted by county using Arc Geographic Information System Pro ( ArcGIS Pro 3.2.2) (open source alternative: QGIS, version 3.36.3) to visualize the geospatial distribution of the disease. An epidemic curve was constructed using the date of symptom onset and mortality for all confirmed cases. We also estimated the death rate among individuals with negative LF test result. We used log binomial regression to assess the association between socio-demographics, clinical characteristics, and mortality. The regression model focused on a group of individuals who have been diagnosed with LF among confirmed LF patients. Models’ results were presented as risk ratios (RR) with 95% confidence intervals (CI) and p-values <0.05 were considered statistically significant.

## Results

### Lassa fever diagnosis, ribavirin treatment, and outcome

**Figure 1.**
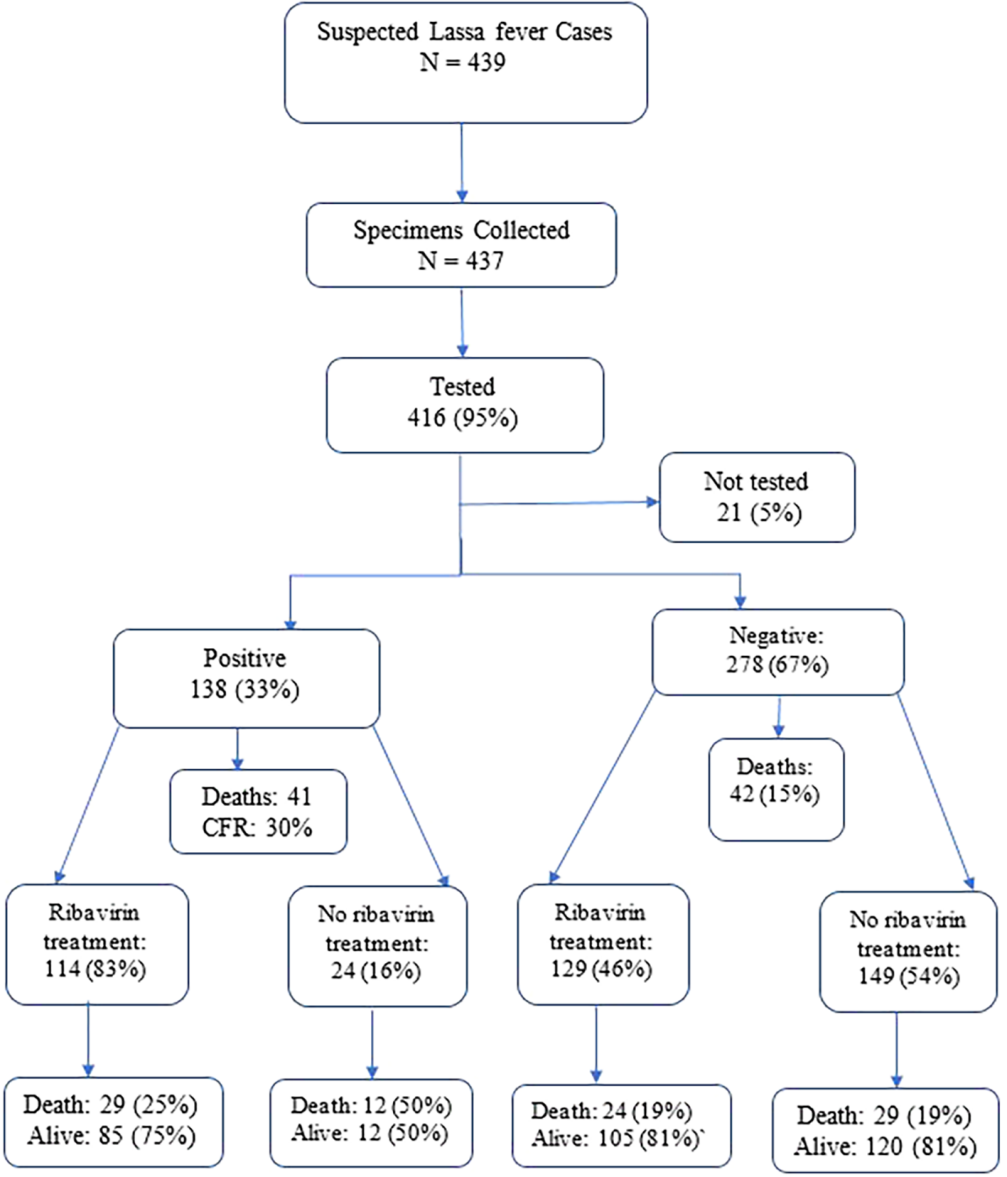
Lassa fever diagnosis, treatment and hospital outcome, Liberia 2022-2023.

A total of 439 suspected LF cases were reported between January 2022 and December 2023 ( Figure 1). The median age was 22 (IQR 10-33) years. The majority (66%, 290/439) of the suspected cases were below 30 years. Women (53%, 233/439) of the cases. Occupation data was missing for 174 participants. Among the 265 with recorded occupations, students constituted 32% (141/439) followed by business persons (12%, 51/439). Geographically, Bong County reported 44% (192/439) of the suspected cases, followed by Grand Bassa County (20%, 89/439) and Nimba County (18%, 80/439) ( Table 1).

**Table 1.** Characteristics and positivity rate for Lassa fever diagnosis using reverse transcription-polymerase chain reaction (RT-PCR), Liberia, 2022-2023.

Characteristics	Total suspected *		PT-PCR tested *		Positivity rate ^¥^	
	n	%	n	%	n	%
**Total**	439		416	95	138	33
Age (years)						
≤14	143	33	133	32	45	33
15-29	147	33	140	34	50	36
30-39	78	18	73	18	23	31
40-49	46	10	45	11	12	27
≥50	25	6	25	6	8	32
Median age	22 (IQR:10-33) Years					
**Gender**						
Male	206	47	206	50	60	29
Female	233	53	233	56	78	33
**Occupation**						
Business	51	11	51	12	15	29
Farmer	33	8	31	7	8	26
Housewife	16	4	16	4	8	50
Student	141	32	137	33	50	36
Tapper	19	4	19	5	11	58
Others ^β^	8	2	8	2	3	38
Unknown	171	39	154	37	13	8
**Reporting county**						
Bong	192	44	171	41	60	35
Grand Bassa	89	20	88	21	45	51
Nimba	80	18	80	19	27	34
Montserrado	56	13	56	13	5	9
Others ^β^	22	5	21	5	1	5

* Number (and column-proportion) for characteristics and RT-PCR tested.

^¥^ Number (and row-proportion) positivity rate).

^β^ Others (number of suspected cases): River Gee (n=5), Grand Kru (n=7), Maryland (n=3), Bomi (n=2), Lofa (n=2), Margibi (n=2), Sinoe (n=1).

### Time from symptom onset to hospital admission

Among the 323 participants with recorded dates, the overall median number of days between symptom onset and admission was 4 (IQR 2-7). Median times by gender were similar (4.5 (IQR: 2-7) vs 4.0 (IQR 1-7) days). When stratified by county, Nimba had the shortest median time, with a median of <1 day (IQR < 1-1.5).

### Positivity rate of Lassa fever cases

Of the 439 individuals suspected of LF, 437 specimens were collected and 95% (416/439) were tested using RT-PCR test and 138 were LF positive. The overall positivity rate was 33% (138/416) ( Figure 1 & Table 1). Among suspected cases with recorded occupations, tappers accounted for 58% (11/19), followed by housewives (50%, 8/16). Grand Bassa County reported 51% (45/88) of the positivity rates followed by Bong (35%, 60/171) and Nimba Counties (34%, 27/80).

### Ribavirin treatment among Lassa fever cases

Ribavirin treatment was administered to 57% (249/439) of hospitalized suspected LF cases ( Figure 1).

**Figure 2.**
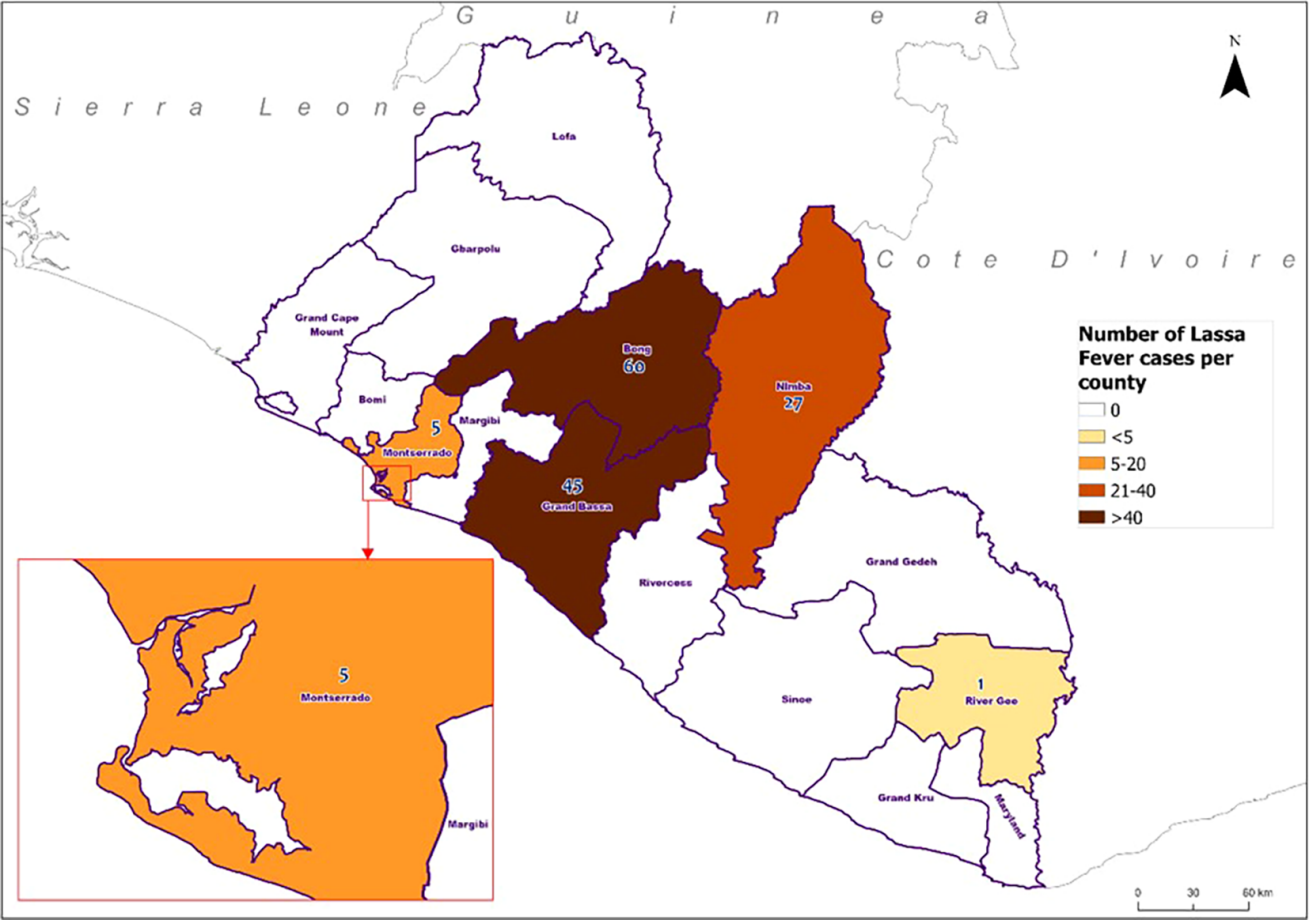
Geographical distribution of confirmed Lassa fever cases by reporting county, Liberia, 2022-2023.

Ribavirin treatment was administered to 83% (115/138) of the confirmed LF cases ( Figure 2).

### Epi-curve of confirmed Lassa fever cases by reporting epi week and year

**Figure 3.**
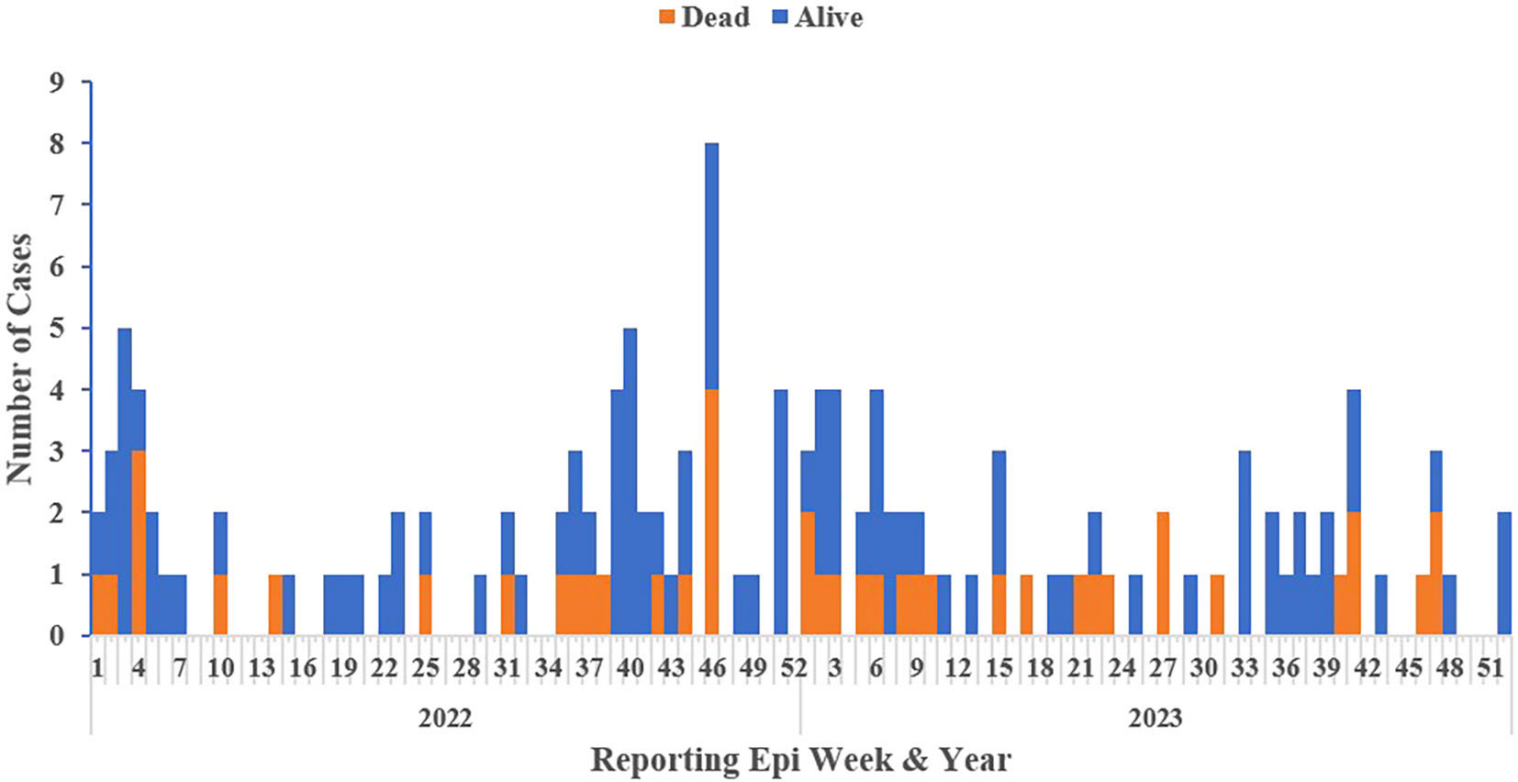
Epi curve of confirmed Lassa fever cases and deaths by reporting week and year, Liberia, 2022-2023.

The 2022 calendar year accounted for over half (52%, 72/138) of the confirmed cases. Majority of the confirmed (59%, 81/138) clustered between epi-weeks 1-12 and 40-52, aligning with the dry season (October – March) ( Figure 3).

### Type of exposure and clinical characteristics of confirmed Lassa fever cases

Rodent exposure accounted for 68% (94/138) among confirmed LF cases while 8% (11/138) of the total confirmed cases had contact with confirmed LF case (epidemiologically linked to a laboratory confirmed case). For clinical characteristics fever accounted for 93% (128/138), followed by malaise 88% (121/138), headache 83% (114/138), and myalgia 83% (114/138). Less than 1% of patients reported hearing loss ( Table 2).

**Table 2.** Exposure and clinical characteristics of confirmed Lassa fever cases, Liberia, 2022-2023 (N=138).

Characteristics	Confirmed N=138	
Type of exposure	n	%
Exposure to rodent	95	69
Contact with confirmed Lf case	11	8
Unknown	32	23
Signs and symptoms (n=138)		
Fever >38 °C	128	93
Malaise	121	88
Myalgia	114	83
Headache	114	83
Sore throat	86	62
Diarrhea	85	62
Red eyes	81	59
Vomiting	77	56
Chest pain	76	55
Nausea	70	51
Cough	55	40
Bleeding	46	33
Swollen face	43	31
Swollen neck	26	19
Hearing loss	1	0.7

### Deaths and case fatality rates

There were 19% (83/439) deaths among hospitalized suspected LF; 42 deaths occurred among 278 individuals who tested negative ( Figure 2). The remaining 41 were among confirmed cases, with a 30% CFR. The CFR varied significantly by age group (p-value=0.006). The highest CFR was observed in individuals aged 40-49 years recorded (67%, 8/12) CFR, followed by those aged ≥50 years (63%, 5/8). Lower CFRs were seen in the ≤14-year-old group (27%, 12/45), the 30-39 year-old group (26%, 6/23), and the 15-29 year-old group (20%, 10/50).

### Factors associated with mortality among confirmed Lassa fever cases

In bivariate analysis, we found that LF patients ≥30 years were 1.9 times more likely to die (cRR=1.9, 95% CI=1.160-3.136, p = 0.011) compared to those < 30 years; LF patients reported from Nimba County were 2.0 times more likely to die (cRR=2.0, 95% CI=1.129-3.771, p=0.019) compared to those reported from Bong County; LF patients who did not receive ribavirin treatment were 1.8 times more likely to die (cRR=1.8, 95% CI=1.083-3.103, p = 0.024) compared to those who received ribavirin treatment. These associations were statistically significant between LF and mortality. In multivariate analysis, none of the variables was associated with LF mortality ( Table 3).

**Table 3.** Hospital outcome and factors associated with mortality among confirmed Lassa fever cases, Liberia, 2022-2023.

Variable	Confirmed (N=138)	Deaths (CFR (%) (N=41)	cRR (95% CI)	P value	aRR (95% CI)	P value
**Age (Yrs)**	N	N (%)				
<30	95	22 (23)	Ref			
>=30	43	19 (44)	1.9 (1.160- -3.136	0.011	1.2 (0.554-2.656)	0.628
**Sex**						
Male	60	20 (33)	1.2 (0.742--2.065)	0.413		
Female	78	21 (27)	Ref			
**Reporting County**						
Bong	60	14 (23)	Ref			
Grand Bassa	45	11 (24)	1.0 (0.526-2.085)	0.895		
Nimba	27	13 (48)	2.0 (1.129- -3.771)	0.019	1.1 (0.357-3.382)	0.868
**Occupation (n=95)**						
Business	15	6 (40)	Ref			
Farmer	8	5 (63)	1.5 (0.688-3.547)	0.286		
Healthcare workers	3	0				
House wife	8	2 (25)	0.6 (0.161-2.412)	0.495		
Student	50	10 (20)	0.5 (0.217-1.148)	0.102	0.6 (0.240-2.020)	0.506
Tapper	11	4 (36)	0.9 (0.335-2.465)	0.851		
**Duration_ onset to admission (n=103)**						
<7 days	59	19 (32)	Ref			
>=7 days	44	33 (75)	0.7 (0.412-1.460)	0.432		

*CFR=case fatality rate;

cRR=crude risk ratio;

aRR=adjusted risk ratio;

CI=confidence interval;

Ref=reference point.

## Discussion

The study described the epidemiological characteristics and fatality among LF cases hospitalized during the 2022-2023 outbreak in Liberia. Findings showed a substantial burden of LF in Liberia during the outbreak with positivity rates (33%) and CFR (30%), and even higher CFR among the elderly population. Younger age groups were disproportionately affected. Furthermore, the dry season coincided with a surge in confirmed cases. Fever, headache and malaise were the most frequently reported symptoms, and rodent exposure was common among the confirmed cases. Surprisingly, there was death (13%) observed among hospitalized individuals without LF, highlighting challenges in diagnosis and LF management.

Similar to other studies, ^6^^,^^18^ the study found increased positivity with notable concentration of cases in Bong, Grand Bassa, and Nimba Counties. These counties are endemic to LF and adjacent to each other, which likely has potential influence on infected individuals migrating across the borders likely due to intermarriages, farming activities, and in search of jobs. Thus, contributing to the spread of LF in these regions. The positivity could be because LF is one of the immediately reportable priority diseases under surveillance in Liberia and an endemic disease. Additionally, strengthened surveillance systems at all healthcare delivery levels with assigned surveillance officers for the detection and reporting of suspected LF cases, a functional testing national public health reference laboratory and, most importantly, the revised 2021 case definition of LF all contributed to improved detection of LF. ^16^

The study highlights that younger age groups are disproportionately affected by LF. This vulnerability is likely to be due to increased exposure to rodent reservoirs. Most confirmed cases reported contact with rodents suggesting exposure to rodent droppings or urine during outdoor activities and food handling. Similar to other studies in Liberia ^6^^,^^18^ and Nigeria, ^19^ majority of the cases were recorded in the dry season (the latter and earlier parts of the two years in the study period: October to March with 2023 accounting for the majority burden. The seasonality of LF cases in West Africa’s dry season is likely due to a combination of factors related to the behavior of the main reservoir, the Mastomys natalensis rodent. During the dry season, with less vegetation and food sources outside, these rodents are likely to seek shelter and food inside homes, increasing their contact with humans. This could lead to frequent interactions between rodents, potentially increasing the spread of the LASV within the rodent population. Less plant cover during the dry season could make rodent burrows and movements noticeable for hunting. A clean environment both in the house and the outside environment might help to reduce the contact between mice and humans. Additionally, effective community infection prevention control programmes in endemic areas by the Ministry of Health and the National Public Health Institute of Liberia might be of help.

Our study identified a high CFR of 33%, more than double the 15% reported by WHO for the region. While this CFR is lower than those reported in Nigeria (60%) ^20^ and Sierra Leone (69%) ^21^ and slightly lower than a previous study in Liberia (40%, 41/103), it remains a significant cause for concern. Furthermore, high CFRs were observed in both treated and untreated patients. The short time between symptom onset and hospital admission suggests that healthcare-seeking behaviors alone cannot fully explain the high CFR. Previous studies have raised concerns about the effectiveness of ribavirin treatment and its potential harm. ^12^ The reliability of the human clinical trial data supporting ribavirin’s use has also been questioned, with pre-clinical studies suggesting that current dosing regimens may not reliably inhibit LASV replication. There was high CFR among middle-aged and older adult population. Although the causes of the high mortality are unknown, it could be likely due to waning immunity among these age groups, making them less effective at fighting off infections like Lassa fever, similar to other studies. ^12^ However, the reasons for this are unclear, warranting further research in understanding the occurrence. Surprisingly, CFR was higher in Nimba, an endemic LF county, a finding similar to what was reported in a previous study. ^6^ The reason for the high CFR in Nimba is unknown considering that Nimba had a median presentation time less than 1 day, pointing out the need for further investigation.

Historical data shows that LF has been misdiagnosed with malaria among patients at treatment facilities in Liberia, which impacts early treatment and patients’ outcome. Suspected LF cases are admitted and begin ribavirin treatment while waiting test results. Some of the LF symptoms (fever and headache) overlap with common diseases like malaria, leading to unnecessary admissions and ribavirin use as well as delays in receiving appropriate treatment. This is concerning, as a 13% death rate was observed among those who did not have LF which may be due to other undiagnosed infections. While the strategy aims to combat LF high CFR, improvements in diagnosis and management are crucial.

These findings have an important implication for the programme’s practice and policy. Addressing the screening and management challenges surrounding LF requires a proactive shift towards preventive measures. Mass vaccination campaigns, although in their developmental stages, offer a promising avenue for mitigating the burden of LF. Initiatives by the WHO and Coalition for Epidemic Preparedness Innovations (CEPI) prioritize LF for vaccine development and enhanced surveillance efforts, ^22^ signaling a step in the right direction. However, given the time frame for vaccine development and implementation, immediate action is warranted. Based on a clearer understanding of seasonal variations within Liberia and across West Africa, public health interventions like vector control programmes and year-round community education campaigns, intensified during the dry season when LF transmission peaks, could significantly enhance community awareness and prevention, thereby reducing mortality rates and easing the burden on healthcare systems. Implementing rodent-proofing measures could likely further reduce exposure risks.

Our findings should be viewed with the following limitations: Firstly, there were incomplete data on duration from onset of symptoms to hospital admission. Exclusion of these records might have affected the estimation of the time from onset of symptoms to hospital admission with respect to socio-demographic characteristics. Secondly, our data was focused on facility-based rather than community-based surveillance, meaning that those who did not seek healthcare were not captured, and we might therefore have underestimated the number of LF cases. Third, we could not explore the effect of ribavirin on mortality due to lack of data. Lastly, our data provides no information on the possible causes of mortality among the suspected cases that were tested negative for LF. Despite these limitations, the study used surveillance data which reflect the program setting, making the findings useful to inform policy and programmes in Liberia and other comparable settings to prevent LF outbreaks.

## Conclusions

This study showed a significant burden of Lassa fever in Liberia during the 2022-2023 outbreak, characterized by positivity, CFR, and death among those without the disease, highlighting the urgent need for proactive prevention measures such as vaccination campaigns and intensive public education. Introducing a rapid point-of-care test for LF would help reduce unnecessary admissions, avoid empirical use of ribavirin, and reduce delays in administering the appropriate treatment. Furthermore, the CFR was high among the middle-aged and older adult population, warranting further investigation. Additionally, the incompleteness of some records highlights the need to strengthen data collection practices within healthcare facilities to ensure complete and accurate data for informed outbreak response efforts, and conduct further research to investigate mortality among suspected cases tested negative for LF.

### Ethics consideration

Access to data for this study was granted by the National Public Health Institute of Liberia. Ethics approvals were obtained from University of Liberia Ethics Review Board on September 27, 2023 (protocol# 23-09-390) and the Ethics Advisory Group for the International Union Against Tuberculosis and Lung Disease, Paris, France, on August 9, 2024; (EAG# 24/23). Informed consent was not obtained as we used routine programme data, which was anonymized by delinking patient identifiers from the dataset.

## Author contributions

“Conceptualization, E. D, R.W.J, B.I.S, H. T, F.T, P. O, M.B, I.F.K, G.W.G, P. K, D. B. L, L.A.E, P. R, S.H; B.T.V., J.S.M.G; methodology, E. D, H.T, P. O, M.B, C.D.U, R.W.J, B.I.S, F. T, P. A, G.W.G,D.B.L, L.A.E, P. R, S. H, B.T.V, P. K, P.A., I.F.K, J.S.M.G; software; validation, E. D, G.W.G; formal analysis, E. D, H.T, M. B, C.D.U, P.A., GEA; investigation; resources; data curation; writing—original draft preparation, E. D, H.T, M. B, writing—review and editing, E. D, H.T, F. T, P.O, C.D.U, B.T.V, M. B, B.I.S, P. K, P. A, L.A.E, P. R, S. H, I.F.K, GEA; visualization, G.E.A, E.D; supervision; project administration; funding acquisition. All authors have read and agreed to the last version of the manuscript.”

## Open access statement

In accordance with WHO’s open-access publication policy for all work funded by WHO or authored/co-authored by WHO staff members, WHO retains the copyright of this publication through a Creative Commons Attribution IGO license ( http://creativecommons.org/licenses/by/3.0/igo/legalcode) which permits unrestricted use, distribution and reproduction in any medium provided the original work is properly cited.

## Data Availability

The dataset used for this study is available at the Division of Infectious Disease and Epidemiology, National Public Health Institute of Liberia and can be accessed upon request in line with the existing data request guide, which provides opportunity for both internal and external data requests ( https://www.nphil.gov.lr/wp-content/uploads/2024/03/nphil-data-request-guide.pdf).
