# Supplementary material for: Epidemiological characteristics and hospital outcomes of hospitalized Lassa fever cases during the 2022-2023 outbreak in Liberia
Source: F1000Res. 2025 Jun 10;13:661. Originally published 2024 Jun 19. [Version 3] doi: 10.12688/f1000research.150743.3 (PMC12150276; doi:10.12688/f1000research.150743.3)
Supplement: Peer review history

# Reviewer report: Reviewer response for version 2

**DOI:** 10.5256/f1000research.181955.r385516

**Published:** 2025-06-04

**Authors:** Qudus Lawal

**Affiliations:** 1 Irrua Specialist Teaching hospital, Irrua, Nigeria

The study is relevant and explores an important aspect of Lassa fever control and prevention. Understanding the epidemiology of Lassa will help improve policies and guidelines.

 The method of retrospective cohort using a national database helps give a reliable estimate.

 A supplementary document of tool used for data collection will be helpful to improve replicability and understanding of what was done

 However, the authors can improve the quality of the article by addressing the following:

 1) Fever, 128 (93%), malaise, 121 (88%), headache, 114 (83%) and myalgia, 114 (83%) were the most common clinical characteristics.

 Comment:    it reads better as Fever, 128 (93%), malaise, 121 (88%), headache, 114 (83%) and myalgia, 114 (83%) were the most common clinical presentation

 2) Age 40-49 years accounted for 8/12 (67%) and those aged≥50 reported 5/8 (63%) of the deaths among the confirmed cases.

 Comment: If the author intends to present the CFR per age group, it should be clearly stated as such.

 Introduction

 3) while a retrospective study in 2022 identified fever, fatigue, sore throat, loss of appetite, headache, vomiting, and myalgia as presenting symptoms among confirmed cases.

 Comment: The findings of the retrospective study didn't contrast the SR rather, it added some other findings, thus the use of 'while' is not appropriate. It will be more important to mention the percentage frequency quoted in these studies, given that it was mentioned for the index study in discussion.

 4) this study aimed to understand the 2022-2023 LF outbreak in Liberia by investigating characteristics of suspected 

 comment: The characteristic of all suspected cases was not fully elucidated. A valuable characteristic will be the final diagnosis of the suspected cases

 5) had a history of rodent contact were also considered suspected cases

 Comment: The author should share how this was determined, was it from historical record, was these captures in the tool used?

 6) epidemiologically linked to a laboratory confirmed case

 Comment: it will be important to know the proportion of diagnosis that were based on these case definition

 7) Individuals with negative results after a RT-PCR testing results were discontinued from ribavirin treatment and were discharged immediately

 Comment: Where were these patients discharged to? Home or hospital, how was their outcome captured

 8) We opted for four sites instead of all eight because these were the sites reporting majority of the cases and reviewing records from these sites would address the incomplete record issue or data quality issues in the existing data set used for the study.

 Comment: This justification is quite unclear. were the incomplete data from the smaller sites. Interestingly the table 1 account for all the sites. Kindly explain further

 9) We performed all statistical analyses using Epi Info analysis software (version 7.2.5.0.) and Stata v18.0 (open-source alternative is R: The R Project for Statistical Computing), version 4.3.2.

 Comment: It would be interesting to know why two statistical package was used for analysis. Which analysis was done with each package

 10A) Figure 1 will be improved if a brief description is given on

                     reason for not testing

                     reason for not treating in positive cases

 10B) for the test negative ribavirin patients, what is the mean/median duration of treatment

 11) Nimba had the shortest median time, with a median of <1 day (IQR < 1-1.5).

 comment: This presupposes that there is a trend to admit within a day of symptoms in this county; it will be important to know the method and unit of measure (minutes/hours/days) that was used to assess this construct.

 12) Positivity rate of Lassa fever cases

 Comment: Positivity rate, as described in this study is a measure of sensitivity of physician suspicion of Lassa (number of suspected cases that tested positive) rather than the distribution of the disease. Using it as a estimate of distribution will be biased as it is more dependent on physician index of suspicion

 13) 68% (94/138) among confirmed LF cases. For clinical characteristics fever accounted for 93% (128/138), followed by malaise (88%, 121/138), headache (83%, 114/138), and myalgia (83%, 114/138).

 Comment: follow a consistent format, percent (fraction)

 14) we found that LF patients ≥30 years were 1.9 times more likely to die

 Comment: It will be good to know what informed the choice of age 30 for categorisation in the bivariate analysis. This is particularly important because in the discussion section, the result categorization used to explain the result was elderly.

 15) Nimba County were 2.0 times more likely to die (cRR=2.0, 95% CI=1.129-3.771, p=0.019) compared to those reported from other counties

 Comment: The comparison is to the ref, (Bongo). Although incidentally, Great Bassa has cRR of 1, which makes the interpretation given by the author accurate

 16) Surprisingly, there was death (13%) observed among hospitalized individuals without LF, highlighting challenges in diagnosis and LF management.

 Comment: This is why it is important to highlight the diagnosis of those who tested negative. Could they be having a diagnosis equally lethal or worse than LF

 17) The high CFR in Nimba could be due to the fact that the county is endemic to LF and sometimes misdiagnosed with malaria, which impacts early treatment among LF patients.

 Comment: This explanation isn't accurate given the study data; median presentation time is less than 1 day

 18) Similar to other studies, the CFR was higher among elderly people, although the causes of high mortality are unknown, we suspect that factors such as comorbidities, waning immunity, etc may have likely contributed. 

 Comment: This should be referenced.

Is the work clearly and accurately presented and does it cite the current literature?

Yes

If applicable, is the statistical analysis and its interpretation appropriate?

Partly

Are all the source data underlying the results available to ensure full reproducibility?

No source data required

Is the study design appropriate and is the work technically sound?

Yes

Are the conclusions drawn adequately supported by the results?

Partly

Are sufficient details of methods and analysis provided to allow replication by others?

Yes

Reviewer Expertise:

Clinical care and research

I confirm that I have read this submission and believe that I have an appropriate level of expertise to confirm that it is of an acceptable scientific standard, however I have significant reservations, as outlined above.

# Reviewer report: Reviewer response for version 2

**DOI:** 10.5256/f1000research.181955.r385015

**Published:** 2025-06-03

**Authors:** Davidson Hamer, Oluwafemi Balogun

**Affiliations:** 1 Department of Global Health, Boston University School of Public Health, Boston, MA, USA; 2 Department of Medicine, Massachusetts General Hospital (Ringgold ID: 2348), Boston, Massachusetts, USA

This article is much improved. There are just a few minor formatting and grammatical issues to address.

 Abstract. Results. Second to last line, need to add a space between aged and ≥50.

 Results. In section entitled "Deaths and case fatality rates" need to add spaces between ages and "year old group" as in "30-39 year old group (26%, 6/23), and the 15-29 year old group".

 Results. In section entitled "Factors associated with mortality....". all the results should be described in the past tense (specifically the word compare should be in the past tense throughout the paragraph).. For example "...found that LF patients ≥30 years were 1.9 times more likely to die (cRR=1.9, 95% CI=1.160-3.136, p = 0.011) compared to those < 30 years;.."

 Last sentence of this section. could be rephrased to "....none of the variables was associated with LF mortality...".

Is the work clearly and accurately presented and does it cite the current literature?

Yes

If applicable, is the statistical analysis and its interpretation appropriate?

Partly

Are all the source data underlying the results available to ensure full reproducibility?

No

Is the study design appropriate and is the work technically sound?

Yes

Are the conclusions drawn adequately supported by the results?

Yes

Are sufficient details of methods and analysis provided to allow replication by others?

Yes

Reviewer Expertise:

Emerging infectious diseases, antimicrobial resistance, malaria, Chagas disease

We confirm that we have read this submission and believe that we have an appropriate level of expertise to confirm that it is of an acceptable scientific standard.

# Reviewer report: Reviewer response for version 1

**DOI:** 10.5256/f1000research.165339.r296578

**Published:** 2024-09-09

**Authors:** Elisabeth Fichet-Calvet

**Affiliations:** 1 Bernhard Nocht Institute for Tropical Medicine, Hamburg, Germany

The study describes cases of Lassa fever (LF) recorded in Liberia over a 2-year period (January 2022-December 2023). It presents socio-demographic variables such as the sex, age, occupation and place of origin of the patients. The clinical picture and the mortality rate complete this description.  These data are important to communicate because the epidemiology of LF is poorly documented in Liberia. I therefore encourage the authors to index these data.

 However, I feel that a major revision of the manuscript and the analyses is essential in order to improve readability and highlight the important points that are currently diluted in the text. 

**General comments:**

 The manuscript is full of percentages; I counted over 200 in the introduction, results, discussion, figures and tables. This is far too many, because in the end you don't know which ones to pick out. There is often redundancy in the figures presented in the text and in the tables. There is no point in repeating the same information twice in a scientific text. It is also important to always mention the denominator when quoting a percentage in the text.

 Percentages are often compared using terms such as "highest", "lower", "more", without any statistics to support the comparison. These formulations should be removed from the manuscript as they are not scientific. Only statistics can confirm that one figure is smaller or larger than another.

 The abbreviations “LF” for Lassa fever and “LASV” for Lassa virus should be used once the term has been quoted in full for the first time. In addition, the lines should be numbered to facilitate the report of the reviewers.

**Specific comments:**

**Introduction:**

- “… disease is primarily transmitted…” should be “LASV is primarily transmitted…” because a disease is never transmitted. It is the pathogen that is transmitted.
- Replace “Mastomys rats” with “the Natal multimammate mice (Mastomys natalensis)”. Use the term of “mice” instead of “rats” throughout the manuscript.
- Transmission of LASV by direct or indirect contact is a hypothesis. It has not been scientifically proven. Therefore, use the conditional tense in this sentence.
- Update the morbidity and mortality figures according to the recent publication done by Basinski,.et.al., 2021 (Ref 1)
- Some case fatality rates are presented; where do they come from? Country? Years?

**Methods:**

- Study design and study setting should be combined in one paragraph. The general setting is useless because the results do not use this information.
- I think the authors did a case-control study, not a cohort study, because they did not analyse, the outcome variable (LF infection) over time. They compared different exposures or variables between cases (LASV positive) and controls (LASV negative).
- The first sentence in “specific setting” should be shortened
- Exposure to LASV: how do you know that patients touched contaminated utensils? What is a rodent-borne food? A rodent contaminated-food? It would be useful to have the questionnaire in a supplementary information in order to know how the authors evaluated/quoted the exposure to rodents.
- “A confirmed case… laboratory confirmed case… or virus isolation)”. This sentence should be removed as the authors only used the RT-PCR to confirm LF in this study.
- CSO and DSO: it is pointless to give abbreviation as this term is not used in the rest of the text.
- Study population: this sentence is redundant with the one in Study design
- Study data sources: also redundant with the information presented at the end of the Specific setting
- Statistical analysis: please use the term of “LASV prevalence” instead of “positivity rate”, and throughout the manuscript. Please specify variables as dependent or independent. Why did not you perform statistics (binomial regression) on LASV prevalence and other variables? You only did statistics on CFR.

**Results**

 Section “Characteristics of the suspected Lassa fever cases”:

- change this title in “Diagnosis, Ribavirine treatment and outcome”

- Figure 1: state the CFR for patients tested negative for LASV.
- The numbers presented in this paragraph are redundant with Table 1. I would remove this part and focus only on LASV-positive patients.

Section “Positivity rate of Lassa fever cases”:

- see my comment above about the positivity rate.

- Use the term of “LASV prevalence” instead of “positivity rate” throughout the text, figure and table.
- Again, there is a lot of redundancy between the text and the table.
- “highest” see my general comment
- Why not to analyse the data to build a model?

Section “Epicurve”:

- look at the “more confirmed cases”: statistics?

Section “Type of exposure”:

- data redundant with table 3, and not well analysed. If you wanted to analyse exposure to rodents, the question would be “are the LASV+ patients more exposed to rodents than the LASV- patients? To do this, you would need to build a model where the RT-PCR result as an outcome (0 or 1) is a function of rodent exposure (absence/presence according to respondent?) + other variables. You did not answer this question in your article.

- I would be curious to know such a result, as I suspect that everyone was exposed to rodents in the area…

Section “Factors associated”:

- this is the most interesting part of the article. However, factors such as “occupation” and “duration of onset prior to admission” are not presented. You could say that these two variables have no effect on CFR when analysed in a bivariate model.

**Discussion:**

- Dry season as risk season: there is no data in this article to support this assumption.

Furthermore, in another article (Dwalu et al. Trend of Lassa fever cases and factors associated with mortality in Liberia, 2016 - 2021: a secondary data analysis. Pan African Medical Journal. 2024;47(22). 10.11604/pamj.2024.47.22.42156) you published data clearly showing Lassa cases throughout the year, in 6 consecutive years between 2016 and 2021. Only two years showed a peak in the dry season. So, I think it is wrong to claim a seasonal risk when your data shows the opposite in 4 out of 6 years.

- Rodent exposure: this is not analysed in this paper (see my comment above).
- High testing: what is that?
- “younger age group more affected”: I have not seen this result. Where did you do the analysis?
- “direct or indirect contact with rodents”: this is not analysed, leading to over-interpretation.
- CFR in other countries: give the raw data, and do not repeat this idea twice at the beginning and in the middle of the discussion.
- 40% (xx/xx?) in Liberia: what is the reference?
- Rodent-proofing measures needs to be proposed with great caution. See our recent paper on rodent control strategies in Guinea (Marien, J., M. Sage, U. Bangura, A. Lame, M. Koropogui, T. Rieger, B. Soropogui, M. Douno, N. Magassouba and E. Fichet-Calvet (2024). "Rodent control strategies and Lassa virus: some unexpected effects in Guinea, West Africa." Emerg Microbes Infect 13(1): 2341141).  
- As you published data from 2016-2021 surveillance in Dwalu et al. 2024, why don’t you discuss the results presented here with those published previously?
- More generally, what is the novelty of this article when compared to Dwalu et al. 2024?

Is the work clearly and accurately presented and does it cite the current literature?

Partly

If applicable, is the statistical analysis and its interpretation appropriate?

No

Are all the source data underlying the results available to ensure full reproducibility?

Partly

Is the study design appropriate and is the work technically sound?

Partly

Are the conclusions drawn adequately supported by the results?

Partly

Are sufficient details of methods and analysis provided to allow replication by others?

Partly

Reviewer Expertise:

Lassa fever, Lassa virus evolution, phylogeography, rodent ecology, West Africa, eco-epidemiology

I confirm that I have read this submission and believe that I have an appropriate level of expertise to confirm that it is of an acceptable scientific standard, however I have significant reservations, as outlined above.

## References

[1] : Bridging the gap: Using reservoir ecology and human serosurveys to estimate Lassa virus spillover in West Africa. *PLoS Comput Biol* .2021;17(3) : 10.1371/journal.pcbi.1008811 e1008811 10.1371/journal.pcbi.1008811 33657095 PMC7959400

# Response

**Published:** 2025-03-21

**Authors:** Emmanuel Dwalu

**Affiliations:** Infectious Disease & Epidemiol, National Public Health Institute of Liberia, Monrovia, Montserrado, Liberia

**The Editor**

**F1000 Peer Review Journal**

**Ref: Response to Peer Reviewers’ Reports - F1000 Research Peer Review: Epidemiological characteristics and hospital outcomes of hospitalized Lassa fever cases during the 2022 – 2023 outbreak in Liberia**

 Dear F1000 Research Editorial Team,

 Thank you for facilitating the peer review process for our manuscript. We have carefully addressed all the reviewers’ comments, and we believe that the revised manuscript now aligns with the journal’s requirements.

 Below, we provide a line-by-line response to the reviewers’ comments, with the original comments highlighted in bold.

 Sincerely,

 Emmanuel Dwalu

**Reviewer # 2:**

**General comments: **

- **The manuscript is full of percentages; I counted over 200 in the introduction, results, discussion, figures and tables. This is far too many, because in the end you don't know which ones to pick out. There is often redundancy in the figures presented in the text and in the tables. There is no point in repeating the same information twice in a scientific text. It is also important to always mention the denominator when quoting a percentage in the text.**
  - We appreciate the reviewer’s observation. We have revised removing some percentages from the introduction section. However, we still think it is necessary to maintain the percentages in the manuscript's results section.
- **Percentages are often compared using terms such as "highest", "lower", "more", without any statistics to support the comparison. These formulations should be removed from the manuscript as they are not scientific. Only statistics can confirm that one figure is smaller or larger than another.**
  - Thanks to the reviewer for this comment. We have revised the manuscript accordingly.
- **The abbreviations “LF” for Lassa fever and “LASV” for Lassa virus should be used once the term has been quoted in full for the first time. In addition, the lines should be numbered to facilitate the report of the reviewers.**
  - We have revised the manuscript accordingly.

**Specific comments:**

**Introduction:**

- **“… disease is primarily transmitted…” should be “LASV is primarily transmitted…” because a disease is never transmitted. It is the pathogen that is transmitted.**
  - We have revised the manuscript accordingly.
- **Replace “Mastomys rats” with “the Natal multimammate mice (Mastomys natalensis)”. Use the term of “mice” instead of “rats” throughout the manuscript.**
  - We agree and have revised the manuscript.
- **Transmission of LASV by direct or indirect contact is a hypothesis. It has not been scientifically proven. Therefore, use the conditional tense in this sentence.**
  - We have revised the manuscript accordingly.
- **Update the morbidity and mortality figures according to the recent publication done by Basinski,.et.al., 2021 (Ref 1)**
  - We appreciate the reviewer for this observation and recommendation. However, the figure presented in the recommended study (a systematic review), has different sources of data but our study (retrospective cohort study) has all the data coming from one surveillance system is Liberia. Therefore, the authors have seen it useful to maintain the figure used in our study.
- **Some case fatality rates are presented; where do they come from? Country? Years?**
  - The authors have cited the reference in the manuscript to reflect ” The case fatality rate (CFR) among hospitalized patients is estimated to be between 15% and 20% [17] but could be more during epidemics [7,18,19][7].

**Methods:**

- **Study design and study setting should be combined in one paragraph. The general setting is useless because the results do not use this information.**
  - Thanks to the reviewer for this comment. In the general setting, we provide an overview of the Liberian health system's structure, with an emphasis on the reference system, which is later linked to LF case definition and case detection in the specific setting. Therefore, we respectfully disagree with the reviewer’s suggestion to combine the design section and remove the general setting.
- **I think the authors did a case-control study, not a cohort study, because they did not analyze, the outcome variable (LF infection) over time. They compared different exposures or variables between cases (LASV positive) and controls (LASV negative).**
  - Thank you for your comment. We would like to clarify that this is a retrospective cohort study (we have now added the word retrospective) in which all suspected Lf cases were followed until diagnosis, and those confirmed with the infection were followed until discharge. The cases (LASV-positive) and controls (LASV-negative) were not selected based on their exposure status.
- **Exposure to LASV: how do you know that patients touched contaminated utensils? What is a rodent-borne food? A rodent contaminated-food? It would be useful to have the questionnaire in a supplementary information in order to know how the authors evaluated/quoted the exposure to rodents.**
  - We agree with the reviewer. There was no questionnaire for data collection, as it was clearly stated that the study obtained and utilized case-based records of routine national surveillance data of all suspected Lassa fever cases reported between January 2022 and December 2023 that were reported to the National Public Health Institute of Liberia and cross-checked. However, in our study methods (specific setting), we have revised the definition of exposure to Lassa virus as “any contact with rodents, either directly (handling rodents or their food sources) or indirectly (through contact with contaminated utensils, feces, urine, or consumption of food contaminated with rodent feces or urine).
  - However, the form had incomplete data which necessitated grouping of the responses into the categories that are reported in the manuscript.
- **“A confirmed case… laboratory confirmed case… or virus isolation)”. This sentence should be removed as the authors only used the RT-PCR to confirm LF in this study. **
  - We have revised the manuscript as: A confirmed case  was defined as a suspected or probable case with a confirmed /positive laboratory test (positive IgM antibody, positive RT-PCR) or epidemiologically linked to a laboratory confirmed case [22].
- **CSO and DSO: it is pointless to give abbreviation as this term is not used in the rest of the text.**
  - We agree and have revised the manuscript.
**            **
- **Study population: this sentence is redundant with the one in Study design**
  - We appreciate the reviewer and have revised the manuscript.
- **Study data sources: also redundant with the information presented at the end of the Specific setting**
  - We appreciate the reviewer’s comment. However, in the entire specific setting, the authors explained LF case detection, recording/documentation and reporting as well as case definition (suspected, probable, and confirmed).  At the end, the authors explained that data from the IDSR ledger and case alert and laboratory paper-based forms were regularly entered into an MS Excel dataset containing the line-list of LF surveillance data in each district, then submitted to the county level and onward to the National Public Health Institute of Liberia.  Information on sources was explained in the succeeding paragraph (Study data sources, variables, and validation).
- **Statistical analysis: please use the term of “LASV prevalence” instead of “positivity rate”, and throughout the manuscript. Please specify variables as dependent or independent. **
  - We have revised the manuscript accordingly.
- **Why did not you perform statistics (binomial regression) on LASV prevalence and other variables? You only did statistics on CFR.**
  - We thank the reviewer for the useful observation. However, the focus of our study was the characteristics and hospital outcomes of Lf patients. We had to indicate the total suspected, tested and positivity to inform our readers before delving into the characteristics of the confirmed and their outcomes. Therefore, the authors think that the analysis performed supports the study objectives' attainment.   

**Results**

**Section “Characteristics of the suspected Lassa fever cases”:**

- **change this title in “Diagnosis, Ribavirin treatment and outcome”**
  - We have revised the manuscript:
  - The CFR for patients tested LASV negative is indicated in figure 2 (15%, 42/278)) against the 30% (41/138) CFR for confirmed cases.
- **The numbers presented in this paragraph are redundant with Table 1. I would remove this part and focus only on LASV-positive patients.**
  - We appreciate the reviewer but the authors think this is relevant to be maintained.

**Section “Positivity rate of Lassa fever cases”: **

- **see my comment above about the positivity rate.**
  - Please see response below response in addition to the succeeding comments.
- **Use the term of “LASV prevalence” instead of “positivity rate” throughout the text, figure and table.**
  - We appreciate the reviewer’s detailed review and comments/recommendations. However, our analysis focused on LASV positivity rather than prevalence. This is because prevalence looks at a specific number of people who have (or had) the disease during a specific time period, which would be the number of truly positive individuals out of the entire population—not just who is tested.
  - The positivity rate—also referred to as “percent positive rate” or “percent positive” is the percentage of all Lf tests performed that are actually positive. Positivity rate is usually expressed as a percentage (Rogers & John Hopkins School of Public Health, 2020).
- **Again, there is a lot of redundancy between the text and the table.**
  - We have revised highlighting the key findings instead of reproducing the table in text.
- **“highest” see my general comment**
  - We agreed and revised the manuscript accordingly.
- **Why not to analyze the data to build a model?**
- The reviewer’s comment is appreciated. However, the authors did not set to build a model in this study. This could be considered in subsequent studies.

**Section “Epicurve”:**

- **look at the “more confirmed cases”: statistics?**

- We have revised the manuscript accordingly.

**Section “Type of exposure”:**

- **data redundant with table 3, and not well analyzed. If you wanted to analyze exposure to rodents, the question would be “are the LASV+ patients more exposed to rodents than the LASV- patients? To do this, you would need to build a model where the RT-PCR result as an outcome (0 or 1) is a function of rodent exposure (absence/presence according to respondent?) + other variables. You did not answer this question in your article.**
- **I would be curious to know such a result, as I suspect that everyone was exposed to rodents in the area…**

- We appreciate the reviewer’s comments. . However, our analysis was focused on describing the characteristics of the confirmed Lf cases and their outcomes. These characteristics included socio-demographics, clinical, and exposures (including rodent exposure, etc). We did not set out to deal with factors associated with LASV positivity among the patients. 

**Section “Factors associated”:**

- **this is the most interesting part of the article. However, factors such as “occupation” and “duration of onset prior to admission” are not presented. You could say that these two variables do not affect CFR when analyzed in a bivariate model. **

- Occupation and duration of onset prior to admission, along with others were presented in the bivariate analysis but were not significant. That’s why we did not present them in the multivariate analysis, but rather focused on those that were significant.

**Discussion:**

- **Dry season as risk season: there is no data in this article to support this assumption.**
- **Furthermore, in another article (Dwalu et al. Trend of Lassa fever cases and factors associated with mortality in Liberia, 2016 - 2021: a secondary data analysis. Pan African Medical Journal. 2024;47(22). 10.11604/pamj.2024.47.22.42156) you published data clearly showing Lassa cases throughout the year, in 6 consecutive years between 2016 and 2021. Only two years showed a peak in the dry season. So, I think it is wrong to claim a seasonal risk when your data shows the opposite in 4 out of 6 years.**

- The reviewer’s comment is appreciated. The two years indicated in the article mentioned above (2019 & 2020) recorded the majority of the cases (54%) but the overall majority was reported during the dry season (79%) covering October to March. Over half of the cases (59%, 81/138) clustered between epi-weeks 1-12 and 40-52, aligning with the dry season (October – March) (Figure 4).” Based on this, the authors could safely say that dry season accounted for the majority of the cases, likewise the current study.
- **Rodent exposure: this is not analyzed in this paper (see my comment above).**
- Rodent exposure was analyzed in table 2 along with clinical characteristics and outcome and 68% of the confirmed cases were exposed to rodents.
- **High testing: what is that? **
- We appreciate the reviewer and have according revised this to high detection and positivity, instead of high testing and positivity rates.
- **“younger age group more affected”: I have not seen this result. Where did you do the analysis?**
- We thank the reviewer for the observation. This analysis can be seen in tables 1 and 3 where 95 (69%) were less than 30 years. 
- However, the authors have agreed to omit this finding from the discussion.
- **“direct or indirect contact with rodents”: this is not analyzed, leading to over-interpretation.**
- Our data analyzed showed exposure to rodents but did not establish direct or indirect contact. This was provided in the discussion to mean that exposure to rodents could be either by direct or indirect contact to rodents. The majority of the confirmed cases reported exposure to rodents suggesting exposure (68%, 95/138).
- We have revised the manuscript to remain with exposure to rodents.
- **CFR in other countries: give the raw data, and do not repeat this idea twice at the beginning and in the middle of the discussion.**
- **40% (xx/xx?) in Liberia: what is the reference?**
- We agree and have revised the manuscript accordingly (40 **(41/103)** **[6]** in Liberia.
- **Rodent-proofing measures need to be proposed with great caution. See our recent paper on rodent control strategies in Guinea (Marien, J., M. Sage, U. Bangura, A. Lame, M. Koropogui, T. Rieger, B. Soropogui, M. Douno, N. Magassouba and E. Fichet-Calvet (2024). "Rodent control strategies and Lassa virus: some unexpected effects in Guinea, West Africa." Emerg Microbes Infect 13(1): 2341141).** 
- We agree with the reviewer and have revised it accordingly **. **
- **As you published data from 2016-2021 surveillance in Dwalu et al. 2024, why don’t you discuss the results presented here with those published previously?**
- **More generally, what is the novelty of this article when compared to Dwalu et al. 2024?**
- The reviewer’s comment is highly appreciated. However, the previous study was focused on trend, though it also highlighted factors associated with mortality. In the current study, clinical and exposure characteristics are highlighted in addition to mortality, which were missing in the previous study. 
- **Is the work clearly and accurately presented and does it cite the current literature?**

**Partly                **

- **Is the study design appropriate and is the work technically sound?**

**Partly**

- **Are sufficient details of methods and analysis provided to allow replication by others?**

**Partly**

- **If applicable, is the statistical analysis and its interpretation appropriate?**

**No**

- **Are all the source data underlying the results available to ensure full reproducibility?**

**Partly**

- **Are the conclusions drawn adequately supported by the results?**

**Partly**

**References**

**1. Basinski AJ, Fichet-Calvet E, Sjodin AR, Varrelman TJ, et al.: Bridging the gap: Using reservoir ecology and human serosurveys to estimate Lassa virus spillover in West Africa.PLoS Comput Biol. 2021; 17 (3): e1008811 PubMed Abstract | Publisher Full Text**

**Competing Interests**

**No competing interests were disclosed.**

**Reviewer Expertise**

**Lassa fever, Lassa virus evolution, phylogeography, rodent ecology, West Africa, eco-epidemiology**

**I confirm that I have read this submission and believe that I have an appropriate level of expertise to confirm that it is of an acceptable scientific standard, however, I have significant reservations, as outlined above.**

# Reviewer report: Reviewer response for version 1

**DOI:** 10.5256/f1000research.165339.r296577

**Published:** 2024-07-19

**Authors:** Davidson Hamer, Oluwafemi Balogun

**Affiliations:** 1 Department of Global Health, Boston University School of Public Health, Boston, MA, USA; 2 Department of Medicine, Massachusetts General Hospital (Ringgold ID: 2348), Boston, Massachusetts, USA

**F1000 Research Peer Review** : Epidemiological characteristics and hospital outcomes of hospitalized Lassa fever cases during the 2022 – 2023 outbreak in Liberia 

Peer Review 

**[General Comment]** 

 Overall, this manuscript provides an insightful epidemiological analysis of the Lassa fever outbreak and its impact on hospitalized patients in Liberia from 2022 to 2023. The authors present compelling findings that address an evolving public health issue in the context of an outbreak. They have adequately described the study setting & population, exposures (sociodemographic and epidemiological characteristics), and outcome measurements.  

 Fundamentally, this manuscript can contribute to the existing literature on Lassa fever by providing insights and understanding into the epidemiological dynamics and clinical outcomes associated with Lassa fever. Additionally, it expands on the substantial burden placed on hospitalized patients during the outbreak, thereby highlighting critical aspects for public health preparedness and response strategies. 

 The title refers to the epidemiological characteristics and hospital outcomes of patients hospitalized with Lassa fever, but the manuscript has a moderate amount of material on the patients who were initially suspected to have Lassa fever but were not confirmed. We recommend focusing the analysis on the confirmed cases and only providing a brief description of the suspected cases at the beginning of the Results.  

**[Specific Comments]**

**Comment 1:** Rather than refer to cases in the title and the abstract, patients is a better term to use (there are sections where the term case is reasonable when summarizing statistics but not when describing the acute infection and outcomes of the pooled cohort of patients.

**Comment 2:** Second sentence of the Background of the abstract, outcomes should be plural as in “...and hospital outcomes of Lassa fever patients hospitalized...”.

**Comment 3:** Abstract, Results. Recommend introducing the abbreviation IQR in the second sentence “...was 22 (interquartile range [IQR] 10-33)...”

**Comment 4:** In the results section of the abstract, the authors mentioned that ***‘290 (69%) of confirmed cases were <30 years.’*** However, in the preceding sentence, the authors stated that there were 138 confirmed cases which equates to 33% positivity rate.  Once you start referring to confirmed cases, the denominator should be all confirmed cases (N = 138) yet the description “290 (69%) of confirmed cases were <30 years old” is incorrect. The next two percentages are based on the correct, smaller denominator.

**Comment 5:** Abstract, Results. Would consider removing mention of the mortality rate among the suspected but not confirmed Lassa fever cases. There is no information provided on the possible causes of febrile illness in these patients so readers will have no idea what they died from (probably a mix of malaria, bacteremia, and potentially other infectious etiologies).

**Comment 6:** Abstract, Results. For the last sentence, please add the denominator for each group (age 40-49 years, age > or = 50 years).

**Comment 7:** Abstract, Conclusions. As suggested above, we would consider removing the phrase “substantial mortality, even among those who tested negative for the virus” since the causes of acute febrile illness in this group are not described. In contrast, it might be useful to mention the empirical use of ribavirin even for patients who were ruled out for Lassa fever, as this means many patients were exposed unnecessarily to a moderately toxic antiviral.

**Comment 8:** Keywords. Suggest removing Liberia and Lassa fever since these are in the article title and all terms in the title will be picked up by search engines. And SORT IT may not be a searchable term. Consider adding ribavirin as a term.

**[Introduction Section]**

**Comment 9:** Mastomys natalensis in the third sentence of the ‘Introduction’ should be written in the standard scientific format as ‘ *Mastomys natalensis’.* This same suggestion applies to the Discussion (third paragraph).

**Comment 10:** Introduction. Although the prevalence of frequently presenting symptoms in suspected Lassa fever cases is interesting, the more relevant symptoms are those demonstrated in patients who have been confirmed.

**Comment 11:** Introduction. Suggest adding risk factors for mortality from Lassa fever as one of the objectives of this analysis.

**[Methods Section]**

**Comment 12:** It will be beneficial to the readers of the journal to know the type of cohort study conducted. Kindly specify it is retrospective or prospective ( **Study Design**).

**Comment 12:** Methods, Specific setting. Third line. Suggest rephrasing to “...for Lassa fever to identify suspect cases..”.

**Comment 13:** In the context of the first and second sentence of the **Specific Setting** of the **Methods section**, please include a phrase/sentence that indicates the reporting tool, means of communication, and timeliness of reporting (window period) employed by the community healthcare workers when reporting suspected cases to the nearest health facility for verification.

**Comment 14:** Although this sentence (In the Study Settings section), ***‘The IDSR case alert and laboratory submission forms were then completed and finally samples were collected’***, is understood by the reviewer, for clarification and non-assumption by readers of the journal, the authors should indicate the specific type of sample that was collected from the patients for Lassa fever testing.

**Comment 15:** Generic drug names should be presented in lower case—ribavirin should not be capitalized.

**Comment 16:** Were there specific selection criteria for the use of ribavirin?

**Comment 17:** Concerning this statement, **‘Daily assessments were performed using charts to monitor the patients’ progress’**, please specify ( **concisely**) the assessments done.

**Comment 18:** Please rephrase the statement ***‘Individuals with negative results were discharged immediately after the Ribavirin treatment was discontinued’*** to something like ***‘Individuals with negative results after a repeat RT-PCR testing were discharged immediately after the ribavirin treatment was discontinued.’*** If this is not what the authors are trying to convey, kindly clarify.

**Comment 19:** Kindly **expound** the phrase ***‘and selected treatment centers’*** in the sentence ***‘Due to incomplete information in some records, we reconciled the data using the county surveillance database and selected treatment centers.’*** Succinctly expand the phrase to provide more context to readers.

**Comment 20:** Statistical analysis. The sentence ***‘We used log binomial regression to assess the association between socio-demographics, clinical characteristics, and case fatality rates’*** should be revised to ***‘We used log binomial regression to assess the association between socio-demographics, clinical characteristics, and mortality.***’ CFR is a population-level indicator, and it appears the authors inadvertently substituted this in place of mortality.

**Comment 21:** Statistical analysis. The term risk ratio should be in lower case.

**[Results Section]**

**Comment 22:** Results. Second sentence. Missing a period at the end of the sentence.

**Comment 23:** Results. The authors revealed that some individuals were excluded in the statement ***‘Among 439 individuals, about one quarter (26%, 116/439) were excluded due to missing data on date of symptom onset or admission’***. Specify what the individuals were excluded from, e.g., from the regression analyses or Table 2. Kindly clarify.

**Comment 24:** Results. The utility of the data in Table 2 is relatively limited. The median time from symptom onset to hospital admission was similar across age groups (by a day or two), gender (half a day), and county (except Nimba). This table could be removed and a sentence or two added to the Results to describe the major findings.

**Comment 25:** In **Table 4,** was there any reason why Bong County was selected as the reference group? Or was the county selected randomly?

**Comment 26:** Results. It would be interesting and potentially informative to compare patients who had confirmed Lassa fever (PCR+) who received ribavirin vs. those who did not receive this antiviral treatment. Are there any significant differences in their clinical or demographic characteristics?

**Comment 27:** **Table 4**, Ribavirin treatment (yes or no) was not included in the bivariable and multivariable models. Historical data and corresponding studies have shown a decrease in mortality among those administered (especially within 6 days from symptom onset). For comprehensiveness, it would be important to explore and demonstrate the associations between the administration of the treatment and mortality. Additionally, the adjusted (multivariable) models with its findings would have more validity if this was also accounted for.

**Comment 28:** **Table 4**. The authors used a reference cutoff of 7 days to assess the association between duration from symptom onset to hospital presentation. However, we recommend the authors to explore the 4 days as the reference cutoff. Earlier in the results section, the authors reported the median symptom-hospital duration to be 4 days with an IQR of 2 to 7. For validity reasons, kindly conduct an exploratory analysis/sensitivity analysis to explore this new cutoff.

**[Discussion Section]**

**Comment 29:** The abbreviation CFR was introduced much earlier in the manuscript. It should be used on the first and third lines of the beginning of the Discussion. Also, we recommend using the term ‘patients’ rather than cases in the first sentence of the Discussion.

**Comment 30:** As noted earlier, the group with suspected Lassa fever who were not confirmed is likely a mixture of different etiological agents. Suggest removing the last sentence of the first paragraph of the Discussion for this reason.

**Comment 31:** The last statement of the second paragraph ***‘Additionally, effective community infection prevention control programmes in endemic areas might be of help’*** is quite vague. The authors (in their recommendation) should kindly be specific in who implements and ensures the effectiveness of the community IPC programmes.

**Comment 32:** In the third paragraph, in the third and second to last lines, might be better to state “A clean environment in both the house and outside environment...”

**Comment 33:** Additional information is required for this statement ***‘Similar to other studies, the CFR was higher among elderly people. However, the reasons for this are unclear, warranting further research in understanding the occurrence.’*** Yes, additional studies are warranted to better understand the pathophysiological mechanism contributing to the high CFR among elderly population. However, the authors should state possible reasons for high CFR being demonstrated among this population, e.g. comorbidities, waning immunity, etc.

**Comment 34:** The statement ***‘The high CFR in Nimba could be due to the fact that the county is endemic for Lassa fever, with the majority of cases detected at the health facility rather than through early community detection, which impacts on early treatment’ *** is a bit conflicting with your earlier report. In the results section, the authors observed that Nimba had the shortest median time (<1 day) from symptom onset to hospital admission. Kindly reconcile the two statements and re-phrase accordingly.

**Comment 35:** We suggest the authors re-phrase this ***statement ‘leading to unnecessary admissions and ribavirin use as well as delayed appropriate treatment’*** to ***‘leading to unnecessary admissions and ribavirin use as well as delays in receiving appropriate treatment.’***

**Comment 36:** The findings of the study highlighted a notable concentration of cases in Bong, Grand Bassa, and Nimba Counties. However, the authors did not provide or suggest possible explanations or hypotheses for these observations. Also, Figure 2 illustrates that these three Counties are adjacent to each other. Therefore, it would be beneficial for readers if the authors could delve into the potential influence of infected individuals migrating across County borders. This exploration could shed light on how population movements might contribute to the spread of Lassa fever in this region, offering valuable insights into disease dynamics and transmission patterns.

**Comment 37:** In the paragraph that describes the need for improved strategies, it might be useful to describe the need for a rapid point-of-care test for Lassa fever since this would be so helpful to triage patients for infection control purposes and treatment (especially if more effective antivirals become available).

**References:**

**Comment 38:** There is no need to include Indian Council of Medical Research in the first citation. 

**Comment 39:** Lassa should be capitalized in the third citation. 

**Comment 40:** Several of the article titles are presented in title case rather than having just the first letter capitalized (e.g., citation 7, 11, 12, and 21). 

**Comment 41:** There is a duplicate of Balogun OO et al. This is presented as reference 7 and 14.

Is the work clearly and accurately presented and does it cite the current literature?

Yes

If applicable, is the statistical analysis and its interpretation appropriate?

Partly

Are all the source data underlying the results available to ensure full reproducibility?

No

Is the study design appropriate and is the work technically sound?

Yes

Are the conclusions drawn adequately supported by the results?

Yes

Are sufficient details of methods and analysis provided to allow replication by others?

Yes

Reviewer Expertise:

Emerging infectious diseases, antimicrobial resistance, malaria, Chagas disease

We confirm that we have read this submission and believe that we have an appropriate level of expertise to confirm that it is of an acceptable scientific standard, however we have significant reservations, as outlined above.

# Response

**Published:** 2025-03-21

**Authors:** Emmanuel Dwalu

**Affiliations:** Infectious Disease & Epidemiol, National Public Health Institute of Liberia, Monrovia, Montserrado, Liberia

**The Editor**

**F1000 Peer Review Journal**

**Ref: Response to Peer Reviewers’ Reports - F1000 Research Peer Review: Epidemiological characteristics and hospital outcomes of hospitalized Lassa fever cases during the 2022 – 2023 outbreak in Liberia**

 Dear F1000 Research Editorial Team,

 Thank you for facilitating the peer review process for our manuscript. We have carefully addressed all the reviewers’ comments, and we believe that the revised manuscript now aligns with the journal’s requirements.

 Below, we provide a line-by-line response to the reviewers’ comments, with the original comments highlighted in bold.

 Sincerely,

 Emmanuel Dwalu

**Reviewer # 1:**

**General Comment:**

**Overall, this manuscript provides an insightful epidemiological analysis of the Lassa fever outbreak and its impact on hospitalized patients in Liberia from 2022 to 2023. The authors present compelling findings that address an evolving public health issue in the context of an outbreak. They have adequately described the study setting & population, exposures (socio-demographic and epidemiological characteristics), and outcome measurements.**

**Fundamentally, this manuscript can contribute to the existing literature on Lassa fever by providing insights and understanding into the epidemiological dynamics and clinical outcomes associated with Lassa fever. Additionally, it expands on the substantial burden placed on hospitalized patients during the outbreak, thereby highlighting critical aspects for public health preparedness and response strategies.**

**The title refers to the epidemiological characteristics and hospital outcomes of patients hospitalized with Lassa fever, but the manuscript has a moderate amount of material on the patients who were initially suspected to have Lassa fever but were not confirmed. We recommend focusing the analysis on the confirmed cases and only providing a brief description of the suspected cases at the beginning of the results.  **

- *We appreciate the reviewer’s observation. However, in practice, suspected cases are initiated LF treatment while awaiting the test result, as timely intervention is crucial. By including both suspected and confirmed cases, we provide a comprehensive understanding of the complexities and real-world challenges faced in diagnosing and managing this disease. Therefore, we believe it is relevant to present both suspected and confirmed cases but went detail with the confirmed results.*

**[Specific Comments]**

**Comment 1: Rather than refer to cases in the title and the abstract, patients is a better term to use (there are sections where the term case is reasonable when summarizing statistics but not when describing the acute infection and outcomes of the pooled cohort of patients. **

- *We have addressed this comment in the manuscript accordingly.*

**Comment 2: Second sentence of the Background of the abstract, outcomes should be plural as in “...and hospital outcomes of Lassa fever patients hospitalized...”. **

- We have updated the sentence.

**Comment 3: Abstract, Results. Recommend introducing the abbreviation IQR in the second sentence “...was 22 (interquartile range [IQR] 10-33)…” **

- *We have added the abbreviation of interquartile range.*

**Comment 4: In the results section of the abstract, the authors mentioned that ‘290 (69%) of confirmed cases were <30 years.’ However, in the preceding sentence, the authors stated that there were 138 confirmed cases which equates to 33% positivity rate.  Once you start referring to confirmed cases, the denominator should be all confirmed cases (N = 138) yet the description “290 (69%) of confirmed cases were <30 years old” is incorrect. The next two percentages are based on the correct, smaller denominator. **

- *We have revised the manuscript with the appropriate frequency and proportion.*

**Comment 5: Abstract, Results. Would consider removing mention of the mortality rate among the suspected but not confirmed Lassa fever cases. There is no information provided on the possible causes of febrile illness in these patients so readers will have no idea what they died from (probably a mix of malaria, bacteremia, and potentially other infectious etiologies).  **

- *We thank the reviewer for the comment. We have revised the subheading to "deaths" instead of "mortality." We retained the presentation of deaths among unconfirmed cases due to the relatively high proportion (42/278, 15%), which warrants further investigation to improve in-hospital outcomes. **We have added this recommendation to our paper to inform further research on Lassa fever in Liberia.***

**Comment 6: Abstract, Results. For the last sentence, please add the denominator for each group (age 40-49 years, age > or = 50 years). **

- *We have addressed this comment accordingly.*

**Comment 7: Abstract, Conclusions. As suggested above, we would consider removing the phrase “substantial mortality, even among those who tested negative for the virus” since the causes of acute febrile illness in this group are not described. In contrast, it might be useful to mention the empirical use of ribavirin even for patients who were ruled out for Lassa fever, as this means many patients were exposed unnecessarily to a moderately toxic antiviral. **

- *As noted in point 5 above, highlighting the relatively high death rates among individuals who tested negative for LF underscores the need to improve the management of suspected cases. Regarding ribavirin treatment, it was not continued for patients who tested negative for Lassa fever ("ruled out for Lassa fever").  To avoid confusion, we have revised the sentence to clearly state that individuals with negative RT-PCR results were discontinued from ribavirin treatment and discharged promptly.*
- *We believe the main challenge regarding unnecessary antiviral exposure lies in diagnostic limitations, as LF symptoms often mimic those of other infections—a point we have discussed in detail.*

**Comment 8: Keywords. Suggest removing Liberia and Lassa fever since these are in the article title and all terms in the title will be picked up by search engines. And SORT IT may not be a searchable term. Consider adding ribavirin as a term. **

- We agree with the reviewer’s recommendation and have added ribavirin to the list of key words.

 
**[Introduction Section]**

**Comment 9: Mastomys natalensis in the third sentence of the ‘Introduction’ should be written in the standard scientific format as ‘Mastomys natalensis’. This same suggestion applies to the Discussion (third paragraph).  **

- *We have revised the manuscript accordingly.*

**Comment 10: Introduction. Although the prevalence of frequently presenting symptoms in suspected Lassa fever cases is interesting, the more relevant symptoms are those demonstrated in patients who have been confirmed. **

- *Thanks to the reviewer for the observations. We have included the symptoms reported by confirmed patients aligning with the study's scope { while a retrospective study in 2022 identified fever, fatigue, sore throat, loss of appetite, headache, vomiting, and myalgia as presenting symptoms among confirmed cases  [6,10]*

**Comment 11: Introduction. Suggest adding risk factors for mortality from Lassa fever as one of the objectives of this analysis. **

- *We have added this objective to the manuscript.*

**[Methods Section]**

**Comment 12: It will be beneficial to the readers of the journal to know the type of cohort study conducted. Kindly specify it is retrospective or prospective (Study Design).**

- *We have specified the type of cohort study as “a retrospective cohort study”.*

**Comment 12: Methods, Specific setting. Third line. Suggest rephrasing to “...for Lassa fever to identify suspect cases….”. **

- ** **
*We have changed detected to “identify”.*

**Comment 13: In the context of the first and second sentence of the Specific Setting of the Methods section, please include a phrase/sentence that indicates the reporting tool, means of communication, and timeliness of reporting (window period) employed by the community healthcare workers when reporting suspected cases to the nearest health facility for verification.   **

- *We have updated the section as follows: At the community level, community health workers identified potential Lassa fever cases using the community/simplified case definition. Potential cases were referred to the nearest health facility for verification using the community trigger and referral form, detailing initial assessment to the nearby health facility. Referrals were made immediately or within 24 hours of identification, with a phone call or written message delivered to the nearby health facility.*

**Comment 14: Although this sentence (In the Study Settings section), ‘The IDSR case alert and laboratory submission forms were then completed and finally samples were collected’, is understood by the reviewer, for clarification and non-assumption by readers of the journal, the authors should indicate the specific type of sample that was collected from the patients for Lassa fever testing. **

- *We have added “whole blood” to indicate the sample types.*

**Comment 15: Generic drug names should be presented in lower case—ribavirin should not be capitalized. **

- *We have revised accordingly.*

**Comment 16: Were there specific selection criteria for the use of ribavirin? “… **

- *Yes, ribavirin was administered only to suspected Lassa fever cases while awaiting laboratory test results and continued for confirmed cases but the treatment was discontinued for individuals with a negative result. We have clarified this in the methods section as” While awaiting the laboratory test results, individuals were isolated and admitted to a treatment center where IV ribavirin treatment was initiated if they were classified as “suspected Lassa fever patients”.*

**Comment 17: Concerning this statement, ‘Daily assessments were performed using charts to monitor the patients’ progress’, please specify (concisely) the assessments done. **

- *We have revised the sentence as follows: Daily assessments were conducted, including routine examinations such as physical exams, vital signs monitoring, malaria tests, and other relevant checks, using charts to track the patients' progress.*

**Comment 18: Please rephrase the statement ‘Individuals with negative results were discharged immediately after the Ribavirin treatment was discontinued’ to something like ‘Individuals with negative results after a repeat RT-PCR testing were discharged immediately after the ribavirin treatment was discontinued.’ If this is not what the authors are trying to convey, kindly clarify. **

- *There was no repeat of the RT-PCR test before discharging the patients. Once the results from the National Public Health Reference Laboratory were negative, the patient was discharged accordingly, and the ribavirin treatment was discontinued.  We have clarified this as follows: Individuals with negative RT-PCR results were discontinued from ribavirin treatment and discharged immediately.*

**Comment 19: Kindly expound the phrase ‘and selected treatment centers’ in the sentence ‘Due to incomplete information in some records, we reconciled the data using the county surveillance database and selected treatment centers.’ Succinctly expand the phrase to provide more context to readers. **

- *We agree with the reviewer and have revised the manuscript as “Due to incomplete information in some patient records submitted at the national level, we reconciled the data using the county surveillance database. We opted for four sites instead of all eight because these were the sites where majority of the cases were reported from and reviewing records from these sites would address the incomplete record issue or data quality issues in the existing data set used for the study.*

**Comment 20: Statistical analysis. The sentence ‘We used log binomial regression to assess the association between socio-demographics, clinical characteristics, and case fatality rates’ should be revised to ‘We used log binomial regression to assess the association between socio-demographics, clinical characteristics, and mortality.’ CFR is a population-level indicator, and it appears the authors inadvertently substituted this in place of mortality. **

- *The reviewer’s observation is highly appreciated and we have revised the manuscript.*

**Comment 21: Statistical analysis. The term risk ratio should be in lower case. **

- *We agree with the reviewer and revised accordingly.*

**[Results Section]**

**Comment 22: Results. Second sentence. Missing a period at the end of the sentence. **

- *We have added the period.*

**Comment 23: Results. The authors revealed that some individuals were excluded in the statement ‘Among 439 individuals, about one quarter (26%, 116/439) were excluded due to missing data on date of symptom onset or admission’. Specify what the individuals were excluded from, e.g., from the regression analyses or Table 2. Kindly clarify.**

- *We have revised the manuscript.*

**Comment 24: Results. The utility of the data in Table 2 is relatively limited. The median time from symptom onset to hospital admission was similar across age groups (by a day or two), gender (half a day), and county (except Nimba). This table could be removed and a sentence or two added to the Results to describe the major findings.**

- *We appreciate the comment/recommendation and have revised the manuscript.*

**Comment 25: In Table 4, was there any reason why Bong County was selected as the reference group? Or was the county selected randomly? **

- *As a common practice in modelling, we statistically selected the county with the highest number of cases to enhance interpretability and statistical power.*

**Comment 26: Results. It would be interesting and potentially informative to compare patients who had confirmed Lassa fever (PCR+) who received ribavirin vs. those who did not receive this antiviral treatment. Are there any significant differences in their clinical or demographic characteristics? **

- *Thanks for the comment. We did not compare patients with confirmed LF (PCR+) who received ribavirin versus those who did not because this was not the focus of our study. However, in our regression analysis, we assessed the effect of ribavirin treatment on outcomes. We have added a sentence that there was no significant difference between those who received treatment and those who did not. We hope this addresses the concern.*

**Comment 27: Table 4, Ribavirin treatment (yes or no) was not included in the bivariate and multivariable models. Historical data and corresponding studies have shown a decrease in mortality among those administered (especially within 6 days from symptom onset). For comprehensiveness, it would be important to explore and demonstrate the associations between the administration of the treatment and mortality. Additionally, the adjusted (multivariable) models with its findings would have more validity if this was also accounted for. **

- *We have included a variable “ribavirin treatment” in the regression analysis*
- *We could not analyze the onset of symptoms and treatment initiation because we do not have the data. We have included it as a limitation.*

**Comment 28: Table 4. The authors used a reference cutoff of 7 days to assess the association between duration from symptom onset to hospital presentation. However, we recommend the authors to explore the 4 days as the reference cutoff. Earlier in the results section, the authors reported the median symptom-hospital duration to be 4 days with an IQR of 2 to 7. For validity reasons, kindly conduct an exploratory analysis/sensitivity analysis to explore this new cutoff.**

- *We appreciate the reviewer’s recommendation. We have revised the manuscript to include (1-3 days, and 4-6 days) for the duration from symptom onset to hospital admission.*

 
**[Discussion Section]**

**Comment 29: The abbreviation CFR was introduced much earlier in the manuscript. It should be used on the first and third lines of the beginning of the Discussion. Also, we recommend using the term ‘patients’ rather than cases in the first sentence of the Discussion.**

- *We have updated the manuscript.*

**Comment 30: As noted earlier, the group with suspected Lassa fever who were not confirmed is likely a mixture of different etiological agents. Suggest removing the last sentence of the first paragraph of the Discussion for this reason. **

- *We appreciate the comment. However, as stated earlier, it is concerning for individuals who are suspected of having Lassa fever, test negative, and yet still die, without a clear understanding of the cause. Therefore, we aimed to use these findings to recommend further research into the causes of death among patients who test negative for Lassa fever which is not reported in literature.*

**Comment 31: The last statement of the second paragraph ‘Additionally, effective community infection prevention control programmes in endemic areas might be of help’ is quite vague. The authors (in their recommendation) should kindly be specific in who implements and ensures the effectiveness of the community IPC programmes. **

- *We totally agree with the reviewer’s comment/suggestion and have specified who does the implementation as “Additionally, effective community infection prevention control programmes in endemic areas by the Ministry of Health and the National Public Health Institute of Liberia might be of help”*

**Comment 32: In the third paragraph, in the third and second to last lines, might be better to state “A clean environment in both the house and outside environment...” **

- *We agree with the reviewer’s comment/suggestion and have revised the manuscript “A clean environment in both the house and the outside environment might help to reduce the contact between rats and humans”.*

**Comment 33: Additional information is required for this statement ‘Similar to other studies, the CFR was higher among elderly people. However, the reasons for this are unclear, warranting further research in understanding the occurrence.’ Yes, additional studies are warranted to better understand the pathophysiological mechanism contributing to the high CFR among elderly population. However, the authors should state possible reasons for high CFR being demonstrated among this population, e.g. comorbidities, waning immunity, etc. **

- *We appreciate the reviewer’s comment and have revised the manuscript.*

**Comment 34: The statement ‘The high CFR in Nimba could be due to the fact that the county is endemic for Lassa fever, with the majority of cases detected at the health facility rather than through early community detection, which impacts on early treatment’ is a bit conflicting with your earlier report. In the results section, the authors observed that Nimba had the shortest median time (<1 day) from symptom onset to hospital admission. Kindly reconcile the two statements and re-phrase accordingly.**

- *We totally agree and appreciate the reviewer for this observation and recommendation. The high CFR in Nimba could be likely due to the fact that cases presenting with fever are sometimes or most times being treated for malaria because of their presentations before suspecting Lassa fever, which impacts treatment outcome among the patients*
- *We have revised the manuscript accordingly*.

**Comment 35: We suggest the authors re-phrase this statement ‘leading to unnecessary admissions and ribavirin use as well as delayed appropriate treatment’ to ‘leading to unnecessary admissions and ribavirin use as well as delays in receiving appropriate treatment.’ **

- *We agree and have accordingly revised the manuscript.*

**Comment 36: The findings of the study highlighted a notable concentration of cases in Bong, Grand Bassa, and Nimba Counties. However, the authors did not provide or suggest possible explanations or hypotheses for these observations. Also, Figure 2 illustrates that these three Counties are adjacent to each other. Therefore, it would be beneficial for readers if the authors could delve into the potential influence of infected individuals migrating across County borders. This exploration could shed light on how population movements might contribute to the spread of Lassa fever in this region, offering valuable insights into disease dynamics and transmission patterns. **

- *We have revised as “These counties are endemic to Lassa fever and adjacent to each other, which likely has potential influence of infected individuals to migrate across the borders due to intermarriages, farming activities, and in search of jobs.  Thus, contributing to the spread of Lassa fever in these regions”*.

 
**Comment 37: In the paragraph that describes the need for improved strategies, it might be useful to describe the need for a rapid point-of-care test for Lassa fever since this would be so helpful to triage patients for infection control purposes and treatment (especially if more effective antivirals become available).**

- *We have recommended a rapid point of care test for Lassa fever.*

**References:**

**Comment 38: There is no need to include Indian Council of Medical Research in the first citation. **

- *We have revised accordingly.*

**Comment 39**: **Lassa should be capitalized in the third citation. **

- *The comment has been acted upon accordingly.*

**Comment 40: Several of the article titles are presented in title case rather than having just the first letter capitalized (e.g., citation 7, 11, 12, and 21). **

- *We have addressed the comment.*

**Comment 41: There is a duplicate of Balogun OO et al. This is presented as reference 7 and 14.**

- *We thank the reviewer for this observation and we have rectified it.*

**Is the work clearly and accurately presented and does it cite the current literature?**

**Yes**

**Is the study design appropriate and is the work technically sound?**

**Yes**

**Are sufficient details of methods and analysis provided to allow replication by others?**

**Yes**

**If applicable, is the statistical analysis and its interpretation appropriate?**

**Partly**

**Are all the source data underlying the results available to ensure full reproducibility?**

**No**

**Are the conclusions drawn adequately supported by the results?**

**Yes**

**Competing Interests**

**No competing interests were disclosed.**

**Reviewer Expertise**

**Emerging infectious diseases, antimicrobial resistance, malaria, Chagas disease**

**We confirm that we have read this submission and believe that we have an appropriate level of expertise to confirm that it is of an acceptable scientific standard, however we have significant reservations, as outlined above.**
